# Type VI secretion system mutations reduced competitive fitness of classical *Vibrio cholerae* biotype

**DOI:** 10.1038/s41467-021-26847-y

**Published:** 2021-11-09

**Authors:** Benjamin Kostiuk, Francis J. Santoriello, Laura Diaz-Satizabal, Fabiana Bisaro, Kyung-Jo Lee, Anna N. Dhody, Daniele Provenzano, Daniel Unterweger, Stefan Pukatzki

**Affiliations:** 1 Department of Medical Microbiology and Immunology, 6-020 Katz Group Centre, University of Alberta, Edmonton, AB T6G 2S2 Canada; 2 Department of Immunology & Microbiology, University of Colorado Denver Anschutz Medical Campus, 13001 E 17th Pl, Aurora, CO 80045 USA; 3 Department of Biology, The City College of New York, 160 Convent Avenue, New York, NY 10031 USA; 4 Mütter Research Institute, The College of Physicians of Philadelphia, Philadelphia, PA 19103 USA; 5 Department of Biology, University of Texas Rio Grande Valley, One West University Blvd, Brownsville, TX 78520 USA; 6 Max-Planck Institute for Evolutionary Biology, August-Thienemann-Straße 2, 24306 Plön, Germany; 7 Institute for Experimental Medicine, Kiel University, Michaelisstraße 5, 24105 Kiel, Germany

**Keywords:** Bacterial evolution, Bacterial genomics, Pathogens, Microbial ecology

## Abstract

The gram-negative bacterium *Vibrio cholerae* is the causative agent of the diarrhoeal disease cholera and is responsible for seven recorded pandemics. Several factors are postulated to have led to the decline of 6th pandemic classical strains and the rise of El Tor biotype *V. cholerae*, establishing the current 7th pandemic. We investigated the ability of classical *V. cholerae* of the 2nd and 6th pandemics to engage their type six secretion system (T6SS) in microbial competition against non-pandemic and 7th pandemic strains. We report that classical *V. cholerae* underwent sequential mutations in T6SS genetic determinants that initially exposed 2nd pandemic strains to microbial attack by non-pandemic strains and subsequently caused 6th pandemic strains to become vulnerable to El Tor biotype *V. cholerae* intraspecific competition. The chronology of these T6SS-debilitating mutations agrees with the decline of 6th pandemic classical strains and the emergence of 7th pandemic El Tor *V. cholerae*.

## Abstract (web summary)

The bacterium *Vibrio cholerae* has caused seven recorded cholera pandemics. The factors responsible for the decline of 6th pandemic classical biotype strains are not well understood. Here, Kostiuk et al. propose that classical strains underwent sequential mutations in type-six secretion system genes that disadvantaged them when confronted with 7th pandemic El Tor biotype strains.

## Introduction

*Vibrio cholerae* is a gram-negative bacterium and the causative agent of the diarrhoeal disease cholera. Throughout modern history, seven cholera pandemics have been reported, all caused by pandemic strains of the O1 serogroup. Understanding how these O1 pandemic strains evolved is hampered by the absence of available strains. Historical strains were not isolated until the germ theory became accepted in the late 1800s. Furthermore, *V. cholerae* colonises soft tissues and is noninvasive. Therefore, historical specimens for DNA extraction are rare. As a result, DNA sequences are available principally from the 6th pandemic (classical biotype strains, 1899–1923) and the ongoing 7th pandemic (El Tor biotype strains, 1961–today). A preserved intestine from an 1849 cholera victim belonging to the Mütter Museum collection in Philadelphia offers unprecedented insights into 2nd pandemic *V. cholerae* (Supplementary Fig. 7)^1^. The generation of a high-resolution genomic sequence allowed us to track the evolution of one *V. cholerae* virulence factor, the type-six secretion system (T6SS)^2^.

The T6SS is a bacterial nanomachine embedded in the envelope of gram-negative bacteria that mediates contact-dependent translocation of effector toxins into neighbouring cells^2^. Contraction of the T6SS outer sheath ejects an inner tube consisting of polymerised Hcp proteins, capped with effector proteins, into adjacent bacteria. These effectors are toxic unless inhibited by cognate immunity proteins in the attacked bacterium. Three distinct genetic loci across two chromosomes encode the T6SS in *V. cholerae*: auxiliary cluster 1, auxiliary cluster 2 and a large cluster, each harbouring an effector-immunity pair: *tseL* & *tsiV1*, *vasX* & *tsiV2* and *vgrG3* & *tsiV3*, respectively. Effector and immunity pairs must have evolved in tandem with a cognate immunity protein for each effector. Each immunity protein protects only against a single cognate effector because cross-resistance has not been observed^3^.

Based on the similarity of TsiV1, TsiV2 and TsiV3 at each locus in 6th and 7th pandemic strains, we initially classified all 6th pandemic strains into the same compatibility group as the AAA-7th pandemic strains^3^. Compatibility groups were named as follows: the first A refers to TsiV1 (Axx), the second A to TsiV2 (xAx) and the third A to TsiV3 (xxA). Divergent alleles at each locus were named in the order of discovery (A, B, C, D, etc.) for the auxiliary 1, 2 or large cluster, respectively, giving rise to 27 currently known compatibility groups in *V. cholerae*^3^. Compatibility groups were further classified into subfamilies to account for distinct TsiV1 amino acid sequences among the same modules: the 6th pandemic classical strains are represented by compatibility groups A_1_A_1_A_1_ (e.g. strain NIH41), A_2_A_1_A_1_ (e.g. strains CA401 and O395) and A_3_A_1_A_1_ (MZO-2); A_4_A_1_A_1_ (e.g. strain A59) was identified in this study. Members of the same compatibility group are immune to or compatible with each other^3^, as homologous immunity proteins define compatibility groups. In contrast, *V. cholerae* strains that do not encode the same effector and immunity genes will compete and are considered to be incompatible. Pandemic strains are subdivided into two biotypes; the seventh pandemic *V. cholerae* O1 El Tor biotype and the sixth pandemic O1 classical biotype, which evolved as distinct lineages^4–8^. The causes that led the 7th pandemic El Tors to replace classical strains as the source of pandemic cholera remains an intense research area. Three main hypotheses support classical biotype displacement: distinct virulence gene regulation, additional virulence factors encoded by El Tor *V. cholerae*, and biotype-specific phenotypic differences^9–11^. For example, El Tor and classical strains regulate ~1 in 8 genes differently, resulting in biotype-specific spatial-temporal expression of the main virulence factors, cholera toxin (CT) and toxin-coregulated pilus (TCP)^9,10^.

Numerous additional virulence factors encoded within El Tor genomes are either absent or differentially regulated in classical *Vibrio cholerae* strains, including the Vibrio seventh pandemic island 1 (VSP-I) and 2 (VSP-II), HlyA, PilE, MSHA and RTX. Phenotypically, the El Tor biotype displays increased biofilm production and motility compared to classical strains^8–11^. Lastly, lytic phages are specific to either of the two biotypes, inviting the hypothesis that phage population fluctuations could have contributed to the biotype replacement^7,12,13^. El Tor strains cause a less virulent form of cholera accompanied by a more extended period of bacterial shedding^14–16^, likely due to the considerable differences between the biotypes described above, thereby benefiting the pathogen by increasing dissemination time^16^.

We investigated differences in the DNA sequences encoding T6SS genes of classical and El Tor biotype *V. cholerae* to improve understanding of how bacterial competition plays in cholera’s pathogenesis. In this work, we examined three aspects of competitiveness across pandemic cholera strains: the ability to engage in T6SS battle with commensal bacteria, compete with non-pandemic strains and compete with other pandemic strains.

## Results

### 2nd and 6th pandemic classical *V. cholerae* encode polymorphic T6SS

**Fig. 1:**
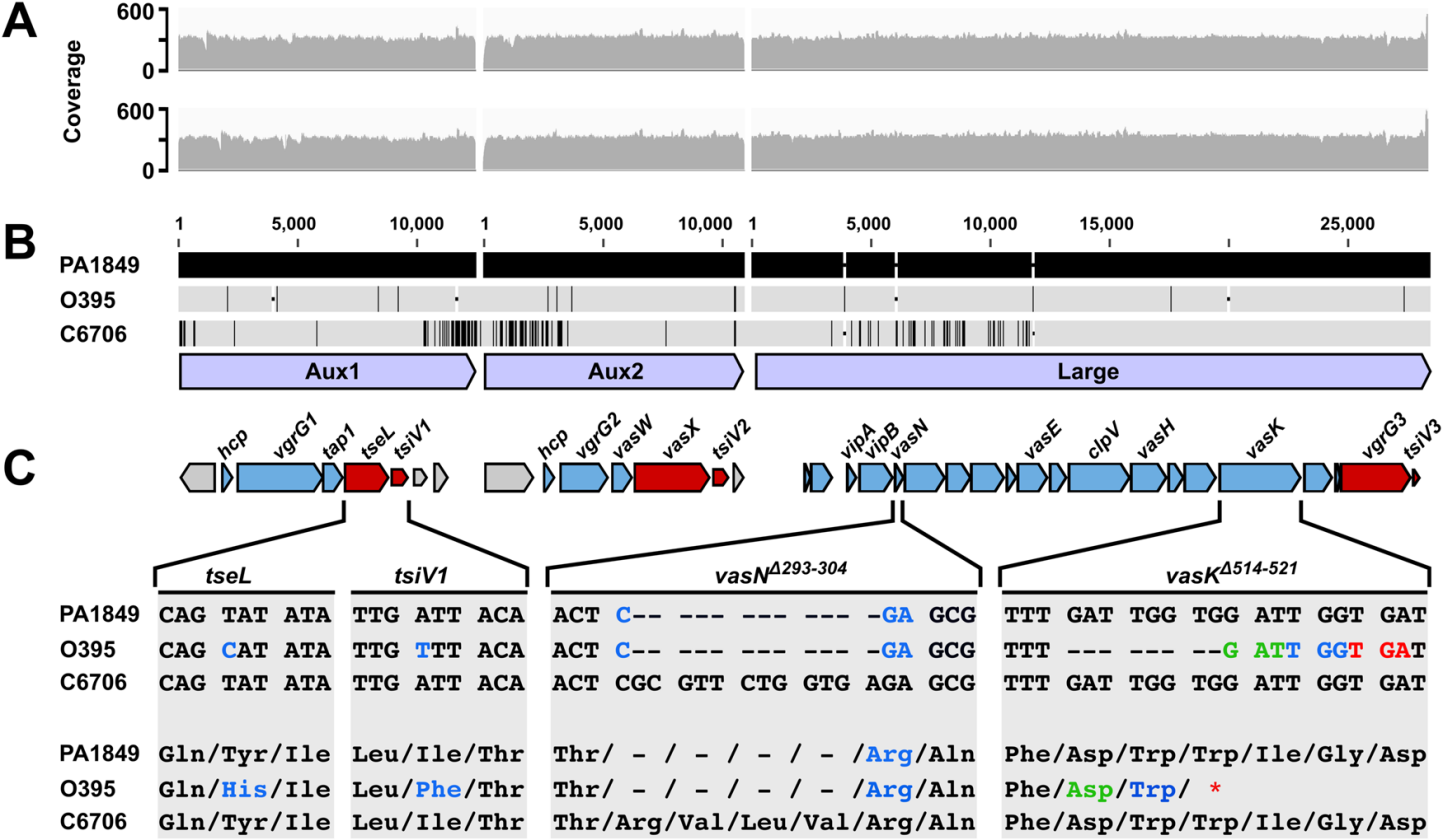
The T6SS gene clusters of the classical *V. cholerae* strain PA1849. **A** Illumina-generated reads were mapped to a 6th pandemic classical strain (O395) and a 7th pandemic El Tor *V. cholerae* strain (C6706). Read coverage plotted against the nucleotide sequence of the three T6SS gene clusters (shown as light blue arrows in panel (**B**)) separated by white vertical lines that indicate the intervening genomic sequences not included in this analysis. The top plot (grey) represents read coverage against the O395 reference, and the bottom plot (grey) represents read coverage against the C6706 reference. **B** Nucleotide alignment of the three T6SS clusters (bottom light blue arrows) from PA1849, O395 and C6706. The top black bars, representing the PA1849 T6SS clusters, are designated as the reference sequences. Conserved residues in O395 and C6706 sequences are represented by grey bars with single-nucleotide polymorphisms (SNPs) highlighted by vertical black lines and insertions or deletions (INDELS) of base pairs represented by gaps with horizontal black dashes. **C** A schematic gene map for each T6SS gene cluster displays structural genes as blue arrows and effector/immunity pairs as red arrows. Zoomed alignments of regions of interest in strains C6706, O395 and PA1849 are shown. Hyphens in the alignment indicate deleted nucleotides (above) and amino acids (below). Nucleotides and their corresponding amino acid changes are coloured. Multiple colours are used to indicate the new reading frame for the *vasK* frameshift mutation. Reads mapping to the *vasN* deletion are shown in the Supplementary material (Supplementary Fig. 10).

The draft genome of the 2nd cholera pandemic classical strain PA1849 isolated from a preserved intestine stored at the Mütter Museum in Philadelphia was recently sequenced^1^. Because the sequencing effort was not designed to provide sufficient sequencing depth to cover the entire T6SS gene clusters, we designed specific booster probes for the enrichment and adequate coverage of these loci. Re-sequencing the microbial content from the same Mütter Museum specimen provided an average coverage depth of ~300x across the T6SS gene clusters (Fig. 1).

An earlier analysis of the three T6SS genetic loci of the 6th pandemic classical *V. cholerae* strain O395 (isolated in India in 1965) revealed frameshift mutations in the large gene cluster^17^. Therefore, we conducted a comparative analysis of the re-sequenced T6SS clusters from the 2nd pandemic strain PA1849 and the 6th pandemic classical strain O395. T6SS sequences from each genome were aligned with those of the El Tor reference strain C6706. The 2nd and the 6th classical pandemic strains PA1849, and O395 encode a 12-nucleotide deletion (Δ293-304) in VCA0109 renamed *vasN*. In addition, the 6th pandemic classical strain O395 harbours an 8-nucleotide frameshift mutation (Δ514-521) in the structural gene *vasK* (VCA0120), one single-nucleotide polymorphism (SNP) in the effector *tseL*, and another SNP in its cognate immunity gene *tsiV1* (Fig. 1). Evaluation of available 6th pandemic classical strain T6SS sequences showed that additional frameshift mutations in the large cluster’s *vipA* (VCA0107) and *vasE* (VCA0114) genes are shared among all 6th pandemic strains (Supplementary Fig. 1). Next, we investigated the impact of these mutations on T6SS activity in classical biotype strains.

### The T6SS of 2nd and 6th pandemic classical strains is disabled

VipA–VipB complexes constitute the contractile outer sheath of the T6SS^18^. Similarly, VasK (TssM in *Escherichia coli*) links the outer membrane protein TssJ with the inner membrane protein TssL, forming the membrane core complex of the T6SS^19^. Deletions of *vipA*, *vasE* or *vasK* in strain V52, which constitutively expresses the T6SS, abolish Hcp secretion (the hallmark of a functional T6SS) and abrogate T6SS-mediated killing^2,20^. Therefore, the mutations highlighted in Fig. 1 and Supplementary Fig. 1 result in truncations of these genes and are prone to disable T6SS activity. Furthermore, the *vasN* in-frame deletion removes four amino acids in VasN, a structural protein essential for Hcp secretion and T6SS function in general^20^.

**Fig. 2:**
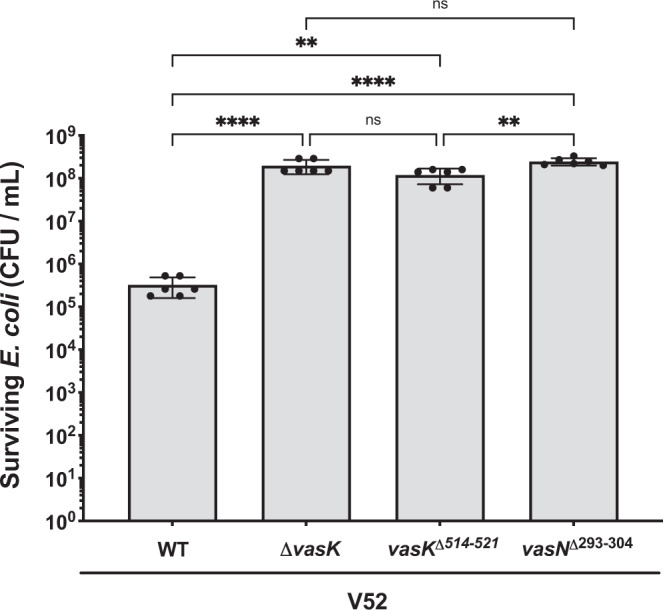
Classical alleles of *vasK* and *vasN* disable the T6SS. A deletion within *vasN* present in 2nd and 6th pandemic strains, or a deletion in *vasK* found in 6th pandemic strains were constructed in *V. cholerae* V52. Strains were assayed for killing *E. coli* MG1655 to determine T6SS function. Wild-type V52 was used as the positive control and V52∆*vasK* as the negative control. Killing assays were performed for 4 h at 37 °C. Mean ± standard deviation of *n* = 3 biologically independent experiments, each performed in duplicate, is shown. Horizontal bars represent the mean and error bars represent the standard deviation. Statistical significance was determined by ordinary one-way ANOVA with Tukey’s multiple comparisons test (****p < 0.0001, **p = (0.0024, 0.0014), ns = not significant). Source data are provided as a Source Data file.

To test whether these polymorphisms restrict the ability of 2nd and 6th pandemic strains to outcompete microbial opponents, we introduced the classical alleles from PA1849 into *V. cholerae* strain V52 with an El Tor T6SS (the V52 T6SS structural components share 99.2% amino acid identity with 7th pandemic El Tor strains such as C6706^17^). This experiment allowed us to determine whether the mutations found in classical strains interfere with T6SS activity while bypassing potential strain-specific regulatory differences^21^. Streptomycin-resistant *V. cholerae* V52 with the *vasK*^*Δ514-521*^ allele from the 6th pandemic classical strains or with the *vasN*^*Δ293-304*^ allele from 2nd and 6th pandemic classical strains were incubated with rifampicin-resistant *E. coli* (see ‘Methods’ for details). The *vasK* ^Δ514-521^ and the *vasN*^*Δ293-304*^ V52 strains lost T6SS activity and did not kill *E. coli* comparably to the *ΔvasK* mutant control (Fig. 2). As reported for previous experiments^21^, wild-type V52 yielded ~1000-fold killing of *E. coli*. The deleted Arginine, Valine, Leucine and Valine residues in the classical *vasN*^Δ293-304^ allele is adjacent to a residue essential for VasN function in *Pseudomonas aeruginosa*^22^ (Supplementary Fig. 2). In *V. cholerae*, these four amino acids are located within a β-strand and are likely to disrupt its secondary structure (Supplementary Fig. 2).

Taken together, these results suggest that the deletion in *vasN* present in both 2nd and 6th pandemic strains disabled the T6SS, permitting the accumulation of additional T6SS-disabling mutations, including a premature stop codon in *vasK* (*vasK*^*Δ514-521*^) in 6th pandemic strains.

### A subset of 6th pandemic classical *V. cholerae* strains is sensitive to T6SS-mediated attack by bacteria belonging to the same compatibility group

**Fig. 3:**
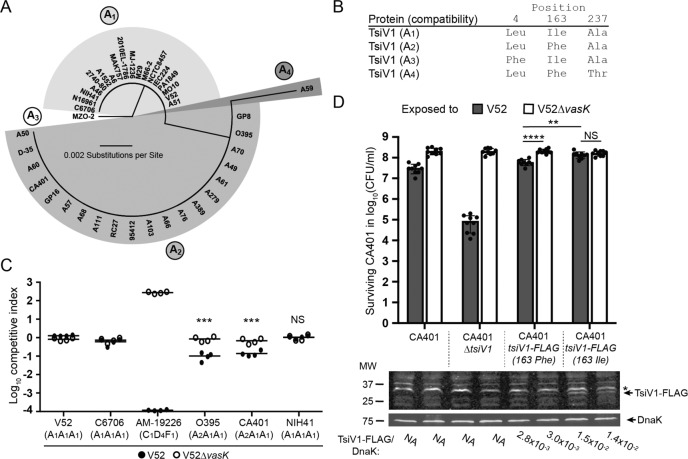
Classical strains harbour multiple amino acid substitutions in TsiV1. **A** Amino acid sequences of TsiV1 cluster into 4 groups (A_1_–A_4_): a radial tree shows the strains that harbour one of four distinct TsiV1 alleles. **B** Overview of the 4 different TsiV1 sequences: the positions and the respective amino acids that distinguish the sequences are shown. The compatibility group representing the TsiV1 immunity protein in the auxiliary cluster 1 is indicated in parentheses. **C** Representative classical strains are sensitive to T6SS-mediated attack by the A_1_A_1_A_1_ strain V52. V52 or V52∆*vasK* were mixed with the indicated strains in a competition assay at a ratio of 1:1 and incubated for 4 h. The competitive index was calculated by dividing the ratio of indicated strain to V52 determined at *t* = 4 h by the ratio determined at *t* = 0 h. The arithmetic mean ± standard deviation of *n* = 2 independent experiments, each performed in duplicate, is shown. Statistical significance was determined by a Student’s unpaired *t*-test (***p = (0.009, 0.013)). **D** TsiV1^163Ile^ protects from a T6SS-mediated attack, whereas TsiV1^163Phe^ does not. V52 was mixed with the indicated strains in a competition assay at a ratio of 1:1 and incubated for 4 h on Lysogeny broth (LB) plates. Mean ± standard deviation of 2 independent experiments, each performed in duplicate, is shown. Statistical significance was determined by a Student’s unpaired *t*-test (****p = 6.5 × 10−6, **p = 0.0013, ns = not significant). Prey bacteria from these experiments were incubated alone under identical conditions and a western blot was performed to detect the production of TsiV1 via an anti-FLAG antibody. The ratio of the signal from the FLAG antibody compared with the DnaK loading control is written underneath the respective lanes. For lanes in which a ratio could not be calculated because TsiV1 is not FLAG-tagged in the respective strains, NA (not applicable) was used instead of a ratio. Source data are provided as a Source Data file.

Compatibility groups comprise *V. cholerae* strains encoding the same T6SS effector and immunity alleles, rendering them immune to reciprocal T6SS-mediated attack and permitting coexistence^3^. Comparative sequence analysis of compatibility groups revealed that TsiV2 and TsiV3 of 6th pandemic strains are identical to their counterparts in C6706 (Supplementary Fig. 3B, C). Comparison of classical TsiV1 amino acid sequences with those of El Tor strains revealed an overall sequence identity of 98.8%. Visualisation of the alignment in a radial tree differentiated four clades of TsiV1 sequences (Fig. 3A). The four polymorphic TsiV1 amino acid sequences vary at positions Leu4Phe, Ile163Phe and Ala237Thr in four different permutations (Fig. 3B) based on single nucleotide substitutions T12G, A487T or G709A in *tsiV1*, giving rise to compatibility groups A_1_–A_4_, respectively.

We compared *V. cholerae* TseL (Supplementary Fig. 3A) to determine whether the polymorphisms observed in TsiV1 amino acid sequences are matched in the cognate effector because immunity protein TsiV1 inhibits TseL effector-mediated T6SS killing^3,23^. The alignment reveals that four TseL alleles are present in the analysed classical *V. cholerae* genomes: 10 out of the 26 strains encode an identical TseL to the allele found in C6706, 14 out of 26 strains differ by a single Tyr528His amino acid substitution, 1 out of 26 differs by both Tyr528His and an additional Ala98Gly substitution and 1 out of 26 is uniquely truncated. Therefore, TseL has undergone sequential mutations over time: no change from PA1849 to CA401, Tyr528His introduced at A57 and the additional Ala98Gly mutation in A279. These results suggest that the mutation of TseL accompanied the mutation of TsiV1 in 6th pandemic strains examined here.

Whether strains with immunity proteins of the same family (e.g. A) but different subfamilies (e.g. A_1_, A_2_) are immune to each other was experimentally determined next. To test the ability of 6th pandemic *V. cholerae* to coexist with other strains of the AAA compatibility family, O395 (A_2_A_1_A_1_), CA401 (A_2_A_1_A_1_) and NIH41 (A_1_A_1_A_1_) were competed against strain V52 (A_1_A_1_A_1_) or V52∆*vasK* (Fig. 3C). T6SS activity of V52 led to an ~10-fold decrease in the number of surviving O395 and CA401 bacteria; in contrast, NIH41 was recovered in equal numbers regardless of exposure to wild-type V52 or V52∆*vasK*, indicating full immunity against T6SS-mediated attack by other members of the A_1_A_1_A_1_ compatibility group. Similar numbers of compatible C6706 (A_1_A_1_A_1_) survived exposure to V52 or V52∆*vasK* resulting in a competitive index of ~1. The competitive index of incompatible AM-19226 (C_1_D_4_F_1_) that survive exposure to V52 was ~10^4^-fold lower, and the competitive index of AM-19226 surviving exposure to V52∆*vasK* was ~10^3^-fold higher (Fig. 3C).

Next, we evaluated TsiV1 immunity gene expression profiles to determine whether the observed sensitivity of A_2_A_1_A_1_ classical *V. cholerae* to T6SS-mediated attack by strains of the A_1_A_1_A_1_ compatibility group is due to transcriptional variation. *TsiV1* expression levels determined by qPCR in select strains did not explain the difference in susceptibility; for example, although O395’s immunity is compromised (Fig. 3C), this strain displayed the highest *tsiV1* transcript levels (Supplementary Fig. 4).

TsiV1 of O395 and CA401 differs from TsiV1 of the fully protected strain NIH41 by a single amino acid substitution, Ile163Phe. We, therefore, replaced *tsiV1* in CA401 with a chromosomally FLAG-tagged copy of the *tsiV1*^*163Phe*^ (A_2_) or *tsiV1*^*163Ile*^ (A_1_) allele to further evaluate the phenotype of this amino acid substitution. The CA401 strain harbouring the *tsiV1*^*163Ile*^ allele was fully protected from the V52 T6SS, while CA401 expressing the *tsiV1*^*163Phe*^ allele experienced a significant reduction in viability analogous to the CA401 WT allele (TsiV1^163Phe^ = A_2_) (Fig. 3D). Deleting *tsiV1* in the same native CA401 background compromised survival by 3 logs as expected (Fig. 3D). Western blot analysis of the two prey strains indicated that CA401 produces approximately 5-fold higher levels of TsiV1^163Ile^ than TsiV1^163Phe^ despite being expressed from the same promoter. This result suggests that the 163Ile El Tor allele is more stable than the classical TsiV1 version or that the classical 163Phe TsiV1 protein is conformationally compromised. Different half-lives may explain why TsiV1 encoded by the A_1_ allele is more protective than the A_2_ version.

### Non-AAA *V. cholerae* outcompete 6th pandemic classical strains

We previously showed that *V. cholerae* belonging to distinct T6SS compatibility groups compete against each other. Those of the AAA genotype are predominately human pathogens that outcompete all other non-AAA strains tested thus far^3^. We hypothesised that 6th pandemic classical strains would be outcompeted by non-AAA strains because of the T6SS disabling deletions in *vasN*^*Δ293-304*^ and *vasK*^*Δ514-521*^ —regardless of their *tsiV1* allele as long the non-AAA strain does not represent an Axx compatibility group (e.g. groups AAB (strain MZO-2) or group ABK (strain YB2A12). Classical 6th pandemic *V. cholerae* were competed against non-AAA environmental isolates DL4211 (CEE), DL4215 (CEC), V51 (CDA) or 1587 (CDC) at a 1:1 ratio. Non-AAA strains outcompeted CA401 and NIH41 between 10^3^ and 10^4^ fold compared to controls (Supplementary Fig. 5). These results indicate that CA401 and NIH41 are sensitive to killing by non-AAA *V. cholerae*, likely placing classical 6th pandemic strains at a disadvantage in intraspecific competition with environmental *V. cholerae*. The subtypes A_1_ and A_2_ do not contribute to a difference in T6SS sensitivity, providing additional support for our compatibility hypothesis.

### Gain, loss and exchange of T6SS mutations in classical *V. cholerae* strains model

Figures 1–3 demonstrate that the T6SS gene clusters of 2nd and 6th pandemic classical strains accumulated mutations affecting their ability to assemble a functional T6SS and, therefore, engage in interbacterial competition. Furthermore, we report that AAA and non-AAA strains outcompete 6th pandemic classical *V. cholerae* tested here.

**Fig. 4:**
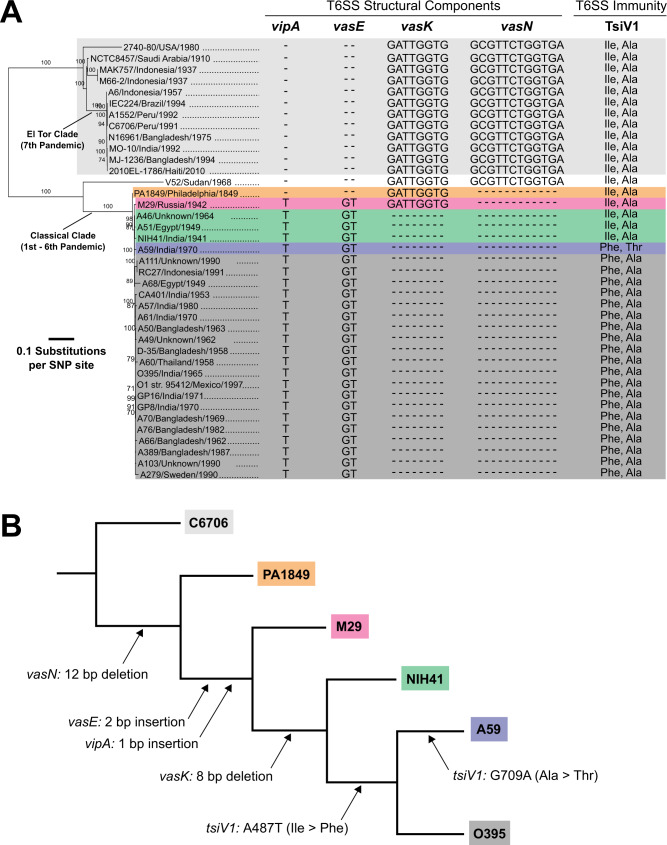
Phylogeny of *V. cholerae* strains and their T6SS gene clusters. **A** Maximum likelihood tree based on core-genome SNP sites of indicated *V. cholerae* strains. Insertions, deletions and nucleotide substitutions are noted. Classical strains with the same mutations in the large cluster of the T6SS and TsiV1 are highlighted in the same colour. The horizontal bars indicate the mean. **B** Schematic representation of the sequential acquisition of T6SS mutations among representative classical *V. cholerae* strains. Arrows highlight the specific changes to the T6SS gene cluster.

Next, we compared available T6SS sequences between classical and El Tor strains with a focus on *vipA*, *vasE*, *vasK* and *vasN*, as well as amino acid substitutions in TsiV1 (Fig. 4A). Comparative sequence analysis of PA1849 and twenty-six 6th pandemic classical strains isolated over 50 years (1940–1990) highlighted how the classical T6SS changed over time throughout multiple pandemics. The 12-nucleotide *vasN* deletion is conserved in all 26 classical strains, and 24 of them also encode the 8-nucleotide deletion in *vasK*. The 2nd pandemic PA1849 and its closest phylogenetic relative, the 6th pandemic M29 strain, retained the wild-type allele of *vasK*. All analysed 6th pandemic *V. cholerae* other than 2nd pandemic PA1849 (i.e. 25 of 26 classical sequences) harboured the additional two nucleotide insertion in *vasE* and one nucleotide insertion in *vipA* (Fig. 4A and Supplementary Fig. 1).

Next, representative strains of each T6SS genotype were chosen to reconstruct the sequential order of mutations acquired within the T6SS gene clusters of classical *V. cholerae* (Fig. 4B). According to this model, the 2nd pandemic strain PA1849 codes only one of the multiple mutations found in 6th pandemic strains, namely the 12-nucleotide deletion in *vasN*. Strain M29 forms an ancestral bridge between PA1849 and the 6th pandemic strains. In addition to the *vasN* mutation, M29 acquired the 2 bp insertion in *vasE* and the 1 bp insertion in *vipA*. Next, all subsequent 6th pandemic classical *V. cholerae* examined here acquired the 8 bp deletion in *vasK*. The nucleotide substitution A487T in *tsiV1* replaced isoleucine with phenylalanine at position 163 of TsiV1, giving rise to strains analogous to CA401 and O395 susceptible to intraspecific T6SS-mediated attack within the same compatibility group, AAA. The additional G709A nucleotide substitution in strains such as A59 gave rise to the Ala237Thr TsiV1 allele.

In summary, our model suggests that classical strains acquired debilitating mutations in their T6SS gene clusters in multiple, sequential steps. Individual mutations were likely inherited vertically from a common ancestor and propagated through the classical population, conferring some form of advantage or, conversely, not having detrimental effects on *V. cholerae* fitness until the emergence of 7th pandemic El Tor strains.

## Discussion

*V. cholerae* of the classical biotype was responsible for the first six reported cholera pandemics. How El Tor strains replaced classical *V. cholerae* as the sole source of contemporary pandemic cholera remains unclear. This study investigated competitive differences between the two biotypes to understand whether the T6SS might have contributed to this replacement. While El Tor strains reach higher cell densities, they express approximately 5-fold less CT and TCP and cause milder disease symptoms than classical *V. cholerae*^24^. We propose that classical *V. cholerae* did not require the T6SS to be successful pathogens through at least five pandemics. Still, they were outcompeted by the emergence of El Tor biotype strains that, despite lower CT and TCP expression, encode additional virulence factors, engage in T6SS-mediated combat and reach higher cell densities.

Pradhan and colleagues reported that in mixed cultures, El Tor strains outnumber classical *V. cholerae* during stationary growth phase and speculated that this phenotype is associated with utilisation of amino acids at higher efficiency in alkaline conditions^25^. Additionally, in contrast to classical *V. cholerae*, growth of El Tor biotype strains is enhanced by the production of 2,3-butanediol, a neutral fermentation end product that prevents the accumulation of organic acids permitting increased El Tor growth rates^8^. These observations suggest that El Tor biotype *V. cholerae* exhibits higher frugality with energetic resources than classical biotype strains, consistent with the view that selective pressure favours pathogens better adapted to capitalise on host resources.

Our work suggests that classical *V. cholerae* lost their ability to employ their T6SS to attack and defend themselves. Consequently, classical strains do not perform effectively in inter- and intraspecific competition with El Tor and other non-classical rivals. Classical strains lost the ability to engage in T6SS attacks as early as the second pandemic, preventing them from killing other prokaryotic cells, and thereby reducing competitiveness compared to *V. cholerae* expressing a functional T6SS.

**Fig. 5:**
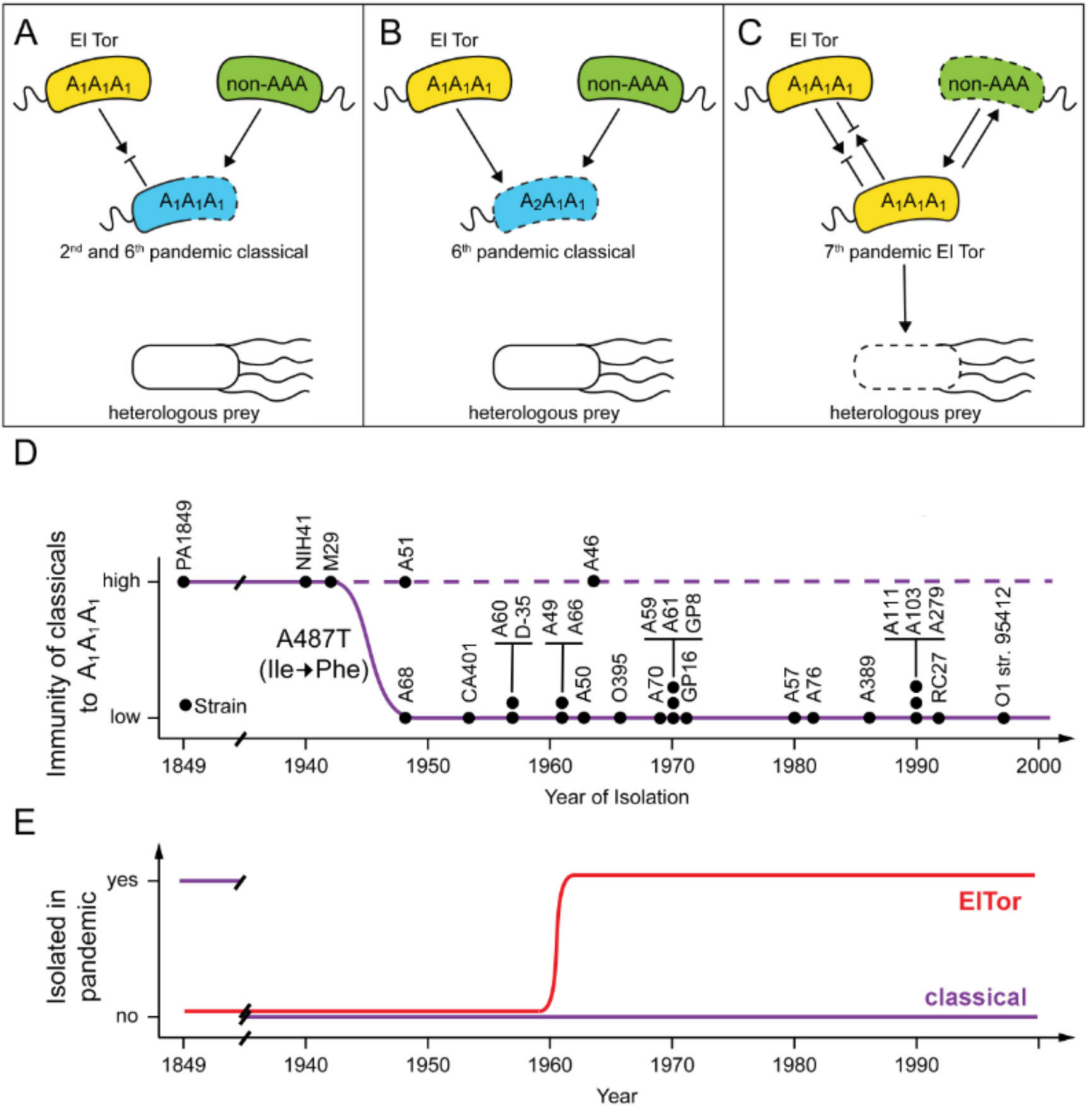
Model for the competitive behaviour of non-classical and classical *V. cholerae* O1 serogroup strains. Panels (**A**–**C**) depict intraspecific T6SS-mediated interactions among *V. cholerae* strains (yellow, azure and green bacteria) and interspecific competition with heterologous pray (white bacterium) not susceptible to killing by any classical pandemic *V. cholerae* due to a disabled *vasN*^Δ293-304^ allele. Classical *V. cholerae* are illustrated as azure bacteria, El Tor biotype are shown in yellow and non-AAA *V. cholerae* are filled in green. Dashed cell outlines represent vulnerability to attack. For clarity, interactions shown here focus exclusively on the compatibility vs vulnerability of pandemic strains (centre). **A** 2nd and early 6th pandemic classical *V. cholerae* bacteria (azure, centre) are sensitive to T6SS-mediated attack (dashed cell outline) by non-AAA *V. cholerae* strains (green, top right). However, compatible immunity proteins protect them against strains belonging to the same compatibility group (continuous cell outline) such as El Tors (yellow, top left). Heterologous prey bacteria are unaffected by 2nd and early 6th pandemic classical *V. cholerae.* **B** Ile163Phe conversion of TsiV1 from subfamily A_1_ to A_2_ leaves 6th pandemic classical *V. cholerae* (azure, centre) vulnerable (dashed cell outline) to T6SS-mediated attack by El Tor (yellow, top left) in addition to non-AAA *V. cholerae* (green, top right) while heterologous prey bacteria remain unaffected. **C** Classical strains are replaced worldwide by 7th pandemic El Tor strains, which are compatible with each other (yellow, top left and centre), engage their T6SS successfully against incompatible non-AAA *V. cholerae* (dashed green cells, top right) and are capable of killing bacterial competitors belonging to other species such as *E. coli* (heterologous prey) depicted below as a white cell. **D** Classical *V. cholerae* strains (filled circles) listed chronologically by year of isolation (*x*-axis) are plotted against immunity to T6SS-mediated attack by A_1_A_1_A_1_ strains (*y*-axis). The nucleotide change from adenosine to thymine at position 487 in *tsiV1* that gave rise to TsiV1^163Phe^ is estimated to have spread among classicals in the 1940s. **E** The 7th cholera pandemic, starting in 1961, is caused by O1 serogroup strains of the El Tor biotype; strains of the classical biotype are only isolated sporadically and have not been involved in pandemics since.

Figure 5A–C presents a model of T6SS-mediated microbial competition for 2nd through 7th pandemic strains hypothesised to occur in any given ecological or host niche^26,27^. The 2nd pandemic strain PA1849 and its closest 6th pandemic relative M29 could not engage their T6SS due to the disabling *vasN*^*Δ293-304*^ deletion. Shared immunity genes protected these early classical strains from attack by pre-7th pandemic El Tor biotype strains^27^ T6SS, but not from environmental (non-AAA) *V. cholerae* (Fig. 5A). Further, TsiV1 Ile163Phe conversion from subfamily A_1_ to A_2_ left 6th pandemic strains sensitive to T6SS-mediated attack by El Tor biotype *V. cholerae* (Fig. 5B). In contrast, the current 7th pandemic El Tor biotype strains are compatible with bacterial kin. Thus, they outcompete environmental (non-AAA) *V. cholerae* and heterologous prey (Fig. 5C)^28^.

Deletions in *vasN* and subsequently *vasK* affect the ability to produce a functional T6SS and predate the single nucleotide substitutions in the immunity gene *tsiV1*. The A_2_ allele of *tsiV1* compromises protection from T6SS-mediated attack by A_1_A_1_A_1_ *V. cholerae* (Fig. 4B). According to this model, in the absence of El Tor strains, selection against 2nd and 6th pandemic classical *V. cholerae*’s inability to engage their T6SS would occur exclusively upon interaction with non-AAA strains (Fig. 5A). One explanation for classical *V. cholerae* having caused five pandemics without an active T6SS may be the existence of a yet-to-be-identified mechanism to outcompete non-AAA competitors during the initial phases of outbreaks when pathogenic *V. cholerae* colonise the small intestine as a mixed population. Subsequently, a short aquatic life phase of classical strains between hosts would further reduce interactions with non-AAA competitors.

Pandemic and environmental strains interpret external cues in the environment and host, adding to the complexity of interbacterial interactions. For example, chitin is required for maximal killing by El Tor strains allowing them to remain competitive against non-AAA *V. cholerae* that are far more likely to express T6SS constitutively during encounters in environmental niches^27^. Conversely, another report demonstrated that intestinal mucins induce expression, and bile acids further modulate the activity of T6SS in pandemic El Tor strains^26^. These reports contribute towards our understanding of the tight regulation T6SS of pandemic strains.

Synergy among *V. cholerae* strains capable of coexisting with other members of their species sharing the same T6SS compatibility group includes sharing exchanged toxins and structural tube proteins^29^. In contrast, toxin exchange between non-kin cells leads to cell death without the possibility to recycle T6SS components. T6SS duelling contributes to kin selection and enhances the ability of kin bacteria to colonise environmental and host niches^3,28^.

The disadvantages of lacking T6SS activity include the inability to battle intra- and interspecific competitors and, consequently, the decreased uptake of exogenous DNA^30–32^, reduced resistance to conjugation^33^ and the failure to deploy anti-eukaryotic effectors^2^. One significant advantage from the loss of T6SS activity among classical strains is the energetic investment that biosynthesis of structural components demands on cell metabolism at the expense of motility and virulence factor production. The absence of Hcp protein synthesis among 6th pandemic *V. cholerae* supports this hypothesis (Supplementary Fig. 6). Lack of T6SS activity may also contribute towards immune avoidance of secreted antigens, thereby facilitating colonisation persistence^24^. Furthermore, a strain unable to engage its T6SS triggers fewer retaliatory events from competing bacteria (a phenomenon known as tit-for-tat), thereby increasing chances for survival in select interspecific encounters^34^.

The loss of T6SS immunity (Fig. 5B) was not selected against until the emergence of El Tor *V. cholerae* (Fig. 5C, E). The deletions that incapacitated T6SS engagement removed the pressure to maintain full immunity among kin strains, permitting the mutations to debilitate T6SS immunity genes without immediate consequence (Fig. 5D). Perhaps not coincidentally, El Tor strains became pandemic after classical *V. cholerae* progressively lost their ability to express the T6SS apparatus and further became susceptible to T6SS-mediated attack by A_1_A_1_A_1_ kin such as those of the El Tor biotype. It is tempting to speculate that these events and other biotype-specific features contributed to the observed reduction in classical strains isolated, as their persistence in environmental niches is hampered by their inability to engage and defend against T6SS attacks by any other *V. cholerae*. Collectively, these factors may have supported the emergence and persistence of El Tor strains as the cause of pandemic cholera (Fig. 5E).

Our study support the idea that intraspecific microbial competition and compatibility among bacterial strains is critical for understanding the succession of pathogenic agents of the same species.

## Methods

### Strains and culture conditions

*Vibrio cholerae* and *E. coli* strains were grown in Lysogeny broth (LB) (1% tryptone, 0.5% yeast extract, 0.5% NaCl) at 37 °C shaking or on LB agar plates (supplemented with 1.5% agar). Bacteria were grown in the presence of 100 µg/mL ampicillin, 100 μg/mL streptomycin or 50 µg/mL rifampicin. The strains used in this study are described in Table 1.

**Table 1:** List of *Vibrio cholerae* strains used in this study and their reference/source^a^.

Strain name	Reference
PA1849	This study
O395	John Mekalanos (Harvard Medical School, Boston, MA, USA)
C6706	^50^
V52	^51^
V52∆*vasK*	^2^
V52*vasK*^∆514-521^	This study
V52*vasN*^∆293-304^	This study
AM-19226	^52^
CA401	Shelley Paine (University of Texas at Austin)
CA401∆*tsiV1*	This study
CA401*tsiV1*163Phe -FLAG	This study
CA401*tsiV*1163Ile-FLAG	This study
NIH41	John Mekalanos (Harvard Medical School, Boston, MA, USA)
DL4211	^28^
DL4215	^28^
V51	Michelle Dziejman (University of Rochester, New York)
1587	^53^

^a^Gene names in italics.

### Molecular cloning

The in-frame deletion mutant CA401∆*tsiV1* was constructed as described previously using pWM91-based knockout constructs^35^. Knock-in versions of PA1849 alleles of *vasK* and *vasN* were made using the same approach. Table 2 lists the primers employed to construct all mutant strains.

**Table 2:** A list of primers used in this study^a^.

Primer name	Sequence
*vasN-*F	TTTATTGCTCTTACTATGCGTAAAGGC
*vasN-*R	CATAGACATTCTTTGGATAAGGCAGC
*vasK-*A	AATACGACTCACTATAGGGCGAATTGGGTAAAGAGGCGTTTCAACTGGC
*vasK-*B	TCAGGACAGATTCATCACCAATCAAACGAGTATGGGTT
*vasK-*C	CTCGTTTGATTGGTGATGAATCTGTCCTGATTGATCCTGA
*vasK-*D	ATTAACCCTCACTAAAGGGAACAAAAGCTGTTGCGTGAAATAGACCGTGG

^a^Gene names in italics.

### Competition assay

Competition assays were performed as described previously^21^. Briefly, two *V. cholerae* strains or a *V. cholerae* strain and *E. coli* MG1655 were mixed at indicated ratios and incubated on pre-dried LB agar plates. After a 4 h incubation at 37 °C, bacteria were harvested, serially diluted and plated onto LB agar plates supplemented with selective antibiotics to enumerate surviving *V. cholerae* or *E. coli* bacteria. The next day, colony-forming units (CFUs) were counted.

### Western blot analysis

Samples for western blots were prepared by growing bacteria in LB to the mid-logarithmic phase of growth, harvesting cells and normalising the OD_600_ to 1.0. Cell pellets from mid-logarithmic growth phase bacterial cultures were resuspended in PBS/2X lysis buffer, boiled and centrifuged. Supernatants were loaded and electrophoresed in either 12% or 15% acrylamide SDS-PAGE at 120 V before transfer to nitrocellulose. After blocking in dry milk/1X TBST for 1 h, blots were probed with primary antibodies diluted to 1:5000 (anti-FLAG), 1:500 (anti-Hcp) or 1:10,000 (anti-DnaK) followed by probing with fluorescently labelled secondary antibodies diluted at 1:10,000. Images were captured with a LICOR Odyssey scanner and analysed using Image Studio Lite software (Supplementary Fig. 11).

### RNA isolation, cDNA synthesis and RT-PCR

RNA from bacterial cultures grown to the mid-logarithmic phase of growth was extracted using TRIzol reagent (Invitrogen), treated with DNase I (Invitrogen) and transcribed into cDNA using the SuperScript III Reverse Transcriptase (Invitrogen). Quantitative real-time PCR (qPCR) was performed with the SensiFAST SYBR No-ROX Kit (FroggaBio) using the CFX96 Real-Time System (Biorad). Thermocycling parameters were as follows: 95 °C for 2 min hot start, 40 cycles at 95 °C for 15 s, and 60 °C for 1 min followed by a melting curve. Primers to genes of interest were designed using PrimerQuest software from Integrated DNA Technologies (IDT). Primers were tested for performance in qPCR with a cDNA concentration gradient, and those with slopes between −3.3 and −3.7, the efficiency of ∼1.0 and *R*^2^ of ∼1.0 were used in the qPCR studies. All primers employed here are listed in Table 2. Expression of *tsiV1* relative to 16 s rRNA control was determined by the 2^−ΔΔCT^ method using the CFX Manager Software (Biorad). The resulting relative quantification (RQ) values of all samples were normalised against expression of strain C6706.

### PA1849 DNA extraction

We analysed tissue collected from a single intestinal sample (specimen 3090.13) taken from a cholera patient in Philadelphia in 1849 and archived by the Mütter Museum in Philadelphia, PA, USA. The Mütter Museum approved sampling and analysing this sample. All tissue handling and library preparation (before indexing amplification) were performed in a positive-pressurised ancient DNA-appropriate laboratory at Arbor Biosciences in which no human or cholera tissue handling or library preparation had previously been performed. We employed field-appropriate standards of anti-contamination technique, including the use of full-body suits, frequent bleach-based work surface and tool decontamination, and blank extraction reactions containing only reagents without template.

Two 5 × 5 mm squares (Supplementary Fig. 7) were cut from the intestinal specimen using decontaminated tweezers and unused razor blades and placed in 2 mL microcentrifuge tubes. An open tube was kept near the processing area during this sub-sampling procedure to serve as another extraction blank.

Each sample was digested with 750 μL solution comprised of 2.5 mM EDTA, 5 mM Tris-Cl, 0.5% SDS, 0.25 ng/μL Proteinase K, which exhibited a final pH of 8.5 after introduction to the dried tissue specimen. After 17 h of incubation at 37 °C, each sample was placed at 4 °C for 4 h, and then 500 μL of the resulting fluid was taken to purification using method B from Glocke & Meyer, 2017^36^, with final eluates of 100 μL TET buffer.

### Library preparation

Roughly 36 ng total tissue DNA extract (15 μL) was taken to library preparation and indexing amplification using the Swift Biosciences (Ann Arbor Michigan) Accel-NGS(R) Methyl-Seq DNA Library Kit following the manufacturer’s standard procedure. The library was index-amplified 10 cycles using dual 8 bp-indexing primers.

### Probe design

Two distinct probe sets were designed for this project, one designed to enrich the entire cholera genome, and another targeting only the three T6SS clusters. The whole-genome kit tiled 80 nt probes every 20 nt across both *V. cholerae* O395 genome reference chromosomes (NCBI Accessions NC_009456 and NC_009457) following soft-masking regions annotated as repeat elements. Where probes overlapped regions of known SNPs in PA1849, probes containing those SNPs were also included (*n* = 203). Any probe candidate that overlapped 20 nt or more with annotated repeat regions, or that had multiple strong hybrid sites in the genome were eliminated from the design. This resulted in a final set of 186,228 probes. A similar procedure was used for the T6SS probe set, starting with the *V. cholerae* T6SS auxiliary 1, auxiliary 2 and large loci as references for probe design, and probes for putative PA1849 SNPs included as well (*n* = 2). This final set comprised 2876 probes.

### Target enrichment

Target enrichment used a combination of whole-genome and T6SS probes at a mass ratio of 99% whole-genome, 1% T6SS or exclusively T6SS probes. Depending on library availability, between 75 and 1000 ng of sample, or 2 and 500 ng of extraction blank library, were taken to enrichment using the myBaits protocol version 3.0. This involved a first round of overnight hybridisation at 60 °C and a single Wash Buffer 2 hot wash, followed by post-capture amplification of 80% of the resulting volume of bead-bound enriched libraries for 14 cycles prior to purification. Then the enriched libraries were taken to a second round of enrichment using identical conditions as the first, except three washes were used during cleanup. Half of the available volume of bead-bound library was taken to 8 cycles of amplification prior to purification. These double-enriched libraries were then submitted for sequencing. Enriched and non-enriched versions of each library were then sequenced using PE100 protocol on a HiSeq 2500 lane, demultiplexed by dual 8 bp barcodes.

### Construction of PA1849 genome

The following analyses were performed on sequencing reads produced with (SL264753) and without (SL264742) target enrichment. Enriched sequencing reads (SL264753) were used to generate consensus sequences and identify variant loci due to significantly increase coverage over the T6SS clusters.

Raw PA1849 FASTQ files were QC validated with FastQC (v0.11.9) to assess Phred quality score and nucleotide distribution over reads. The forward adapter (AGATCGGAAGAGCACACGTCTGAACTCCAGTCA) and the reverse adapter (AGATCGGAAGAGCGTCGTGTAGGGAAAGAGTGT) were removed from paired-end read files, and ends were trimmed with a Phred quality score cut-off of 30 using cutadapt (v1.16). Reads below a minimum length cut-off of 30 nucleotides were removed. Overlapping paired-end reads from the trimmed FASTQ files were merged into single-end reads with NGmerge (v0.2)^37^. Merged reads were aligned to a reference FASTA with the Burrows-Wheeler Aligner (bwa v0.7.17) MEM algorithm^38^. For analysis of the T6SS loci, reference FASTA files were constructed by concatenating genomic islands extracted from C6706 (PacBio sequence of laboratory stock) or O395 (PacBio sequence of laboratory stock) with 100 nucleotide spacers between each region. For phylogenetic analysis, reads were mapped to the whole-O395-genome. SAM files produced were then sorted and marked for duplicates with Picard Tools (v2.17.11) to generate sorted, unique BAM files, which were indexed with Picard Tools^39^. Variants were called against the constructed reference FASTA, and consensus sequences were generated with Pilon (v1.22)^40^. For analysis of the T6SS loci, PA1849 consensus sequences were aligned to C6706 and O395 reference sequences using the progressive MAUVE algorithm (v1.1.1) algorithm in Geneious (v2019.0.4), assuming colinear genomes. Genome coverage plots were generated from sorted, unique BAM files uploaded to the Integrative Genomics Viewer (v2.9.4).

### Assessing damage profile of PA1849 reads

Mapped reads from the sorted, unique BAM files were assessed for fragment length distribution (Supplementary Fig. 8). The median fragment length of >150 bp is higher than the 40 bp length from Devault et al.^1^, because of a difference in Illumina chemistry, lack of sonication of the tissue and enrichment with our updated T6SS probes. Nucleotide substitution patterns characteristic of ancient DNA samples (Supplementary Fig. 9) with mapDamage (v2.0)^41^. Canonical damage patterns typical of ancient DNA (C > T substitutions at the 5′ fragment end and G > A substitutions and the 3′ fragment end) were not observed for these samples. A low frequency of C > T substitutions can be seen at either end of the mapped reads (Supplementary Fig. 9).

### Core-genome phylogeny and genomic alignments

Core-genome-based phylogenetic trees were constructed as follows: Genomic FASTA files for tree building were obtained from the PATRIC database or NCBI (Table 3) and annotated using Prokka (v1.12)^42^. A core genome was extracted from Prokka-output GFF3 files using Roary (v3.11.2)^43^ with a minimum blastp percentage identity of 95% for calling core genes. Roary extracted and aligned 688 core genes (present in >99% of analysed strains). The core-genome alignment was reduced to only loci harbouring polymorphisms using SNP sites (v2.4.1)^44^. A total of 6,225 distinct SNP sites were extracted from the 688 core genes. A Maximum Likelihood phylogenetic tree was built using the RAxML (v8.2.12) GTR model. Model was selected using jModelTest (v0.1.6)^45^. Statistical branch support was obtained from 100 bootstrap repeats. Phylogenetic trees were visualised from RAxML-generated newick files using TreeGraph 2 (v2.15.0-887 beta)^46^. Branches with bootstrapping support values <70 were collapsed. Alignments of amino acid sequences were performed with MUSCLE (v3.8.425)^47^ and alignments of the nucleotide sequences of whole-gene clusters were performed using the progressive MAUVE algorithm^48^ in Geneious (Geneious Prime v2019.0.4). To display the diversity of amino acid sequences as a radial tree, aligned sequences were analysed using RAxML (nucleotide model: GTR Gamma, Algorithm: Rapid hill-climbing)^49^ and displayed with Geneious Prime (v2019.0.4). Some genomes were poorly assembled over the T6SS clusters, and thus corresponding read files from SRA (Table 3) were aligned to the O395 and C6706 T6SS cluster reference sequences in Geneious Prime (v2019.0.4) to determine consensus sequences and identify polymorphisms.

**Table 3:** A list of genomes and reads used in this study.

Strain	Accession	SRA accession
2740-80	GCA_001683415.1	NA
NCTC 8457	GCA_000153945.1	NA
M66-2	GCA_000021605.1	NA
MAK 757	GCA_000153865.1	NA
A6	GCA_001255575.1	NA
IEC224	GCA_000250855.1	NA
A1552	GCA_002892855.1	NA
C6706	GCA_013085075.1	NA
N16961	GCA_000006745.1	NA
MO10	GCA_000152425.1	NA
2010EL-1786	GCA_000166455.2	NA
MJ-1236	GCA_000022585.1	NA
V52	GCA_000167935.2	NA
PA1849	NA	PRJNA767369
M29	GCA_000709105.1	NA
A46	GCA_001259555.1	ERR018132
A51	GCA_001253435.1	ERR018141
A59	GCA_001254535.1	ERR018139
A68	GCA_001259635.1	ERR018135
A111	GCA_001253495.1	ERR018155
RC27	GCA_000176395.1	NA
D-35	GCA_000961975.1	NA
A57	GCA_001250255.1	ERR018140
A61	GCA_001250935.1	ERR018137
A49	GCA_001253835.1	ERR018134
A50	GCA_001254735.1	ERR018142
A60	GCA_001248195.1	ERR018138
O395	GCA_000016245.1	NA
O1 str. 95412	GCA_000348105.2	NA
GP8	GCA_001253575.1	NA
GP16	GCA_001251495.1	NA
A70	GCA_001248905.1	ERR018133
A76	GCA_001259495.1	ERR018143
A66	GCA_001260915.1	ERR018136
A389	GCA_001259795.1	ERR018178
A103	GCA_001254575.1	ERR018145
A279	GCA_001253555.1	ERR018173

### Reporting summary

Further information on research design is available in the Nature Research Reporting Summary linked to this article.

## Supplementary information

Supplementary information.

Reporting summary.

## Data Availability

The raw reads for the PA1849 T6SS gene clusters are available in the NCBI SRA database under the BioProject accession number PRJNA767369. The data generated in this study are provided in the Supplementary information/Source Data file. Source data are provided with this paper.

## Source data

Source Data.

## References

[1] Devault AM (2014). Second-pandemic strain of *Vibrio cholerae* from the Philadelphia cholera outbreak of 1849. N. Engl. J. Med..

[2] Pukatzki S (2006). Identification of a conserved bacterial protein secretion system in *Vibrio cholerae* using the Dictyostelium host model system. Proc. Natl Acad. Sci. USA.

[3] Unterweger D (2014). The *Vibrio cholerae* type VI secretion system employs diverse effector modules for intraspecific competition. Nat. Commun..

[4] Jonson G, Sanchez J, Svennerholm A-M (1989). Expression and detection of different biotype-associated cell-bound haemagglutinins of *Vibrio cholerae* O1. J. Gen. Micro.

[5] Nair GB (2002). New variants of *Vibrio cholerae* O1 biotype El Tor with attributes of the classical biotype from hospitalised patients with acute diarrhea in Bangladesh. J. Clin. Microbiol..

[6] Richardson K, Michalski J, Kaper JB (1986). Hemolysin production and cloning of two hemolysin determinants from classical *Vibrio cholerae*. Infect. Immun..

[7] Takeya K, Otohuji T, Tokiwa H (1981). FK phage for differentiating the classical and El T or groups of *Vibrio cholerae*. J. Clin. Microbiol..

[8] Yoon SS, Mekalanos JJ (2006). 2, 3-Butanediol synthesis and the emergence of the *Vibrio cholerae* El Tor biotype. Infect. Immun..

[9] Jonson G, Svennerholm A-M, Holmgren J (1990). Expression of virulence factors by classical and El Tor *Vibrio cholerae* in vivo and in vitro. FEMS Microbiol. Lett..

[10] Beyhan S, Tischler AD, Camilli A, Yildiz FH (2006). Differences in gene expression between the classical and El Tor biotypes of *Vibrio cholerae* O1. Infect. Immun..

[11] Dziejman M (2002). Comparative genomic analysis of *Vibrio cholerae*: genes that correlate with cholera endemic and pandemic disease. Proc. Natl Acad. Sci. USA.

[12] Zahid MSH (2011). An experimental study of phage mediated bactericidal selection & emergence of the El Tor *Vibrio cholerae*. Indian J. Med. Res..

[13] Faruque SM, Mekalanos JJ (2012). Phage-bacterial interactions in the evolution of toxigenic *Vibrio cholerae*. Virulence.

[14] Bart KJ, Huq Z, Khan M, Mosley WH (1970). Seroepidemiologic studies during a simultaneous epidemic of infection with El Tor Ogawa and classical Inaba *Vibrio cholerae*. J. Infect. Dis..

[15] Khan, M. & Shahidullah, M. *Cholera Due to the El Tor Biotype Equals the Classical Biotype in Severity and Attack Rates* (ICDDR,B, Dacca, 1979). 7365863

[16] Woodward WE, Mosley WH (1972). The spectrum of cholera in rural Bangladesh II. Comparison of El Tor Ogawa and classical Inaba infection. Am. J. Epidemiol..

[17] Miyata ST (2010). The *Vibrio cholerae* type VI secretion system: evaluating its role in the human disease cholera. Front. Microbiol..

[18] Kudryashev M (2015). Structure of the type VI secretion system contractile sheath. Cell.

[19] Durand E (2015). Biogenesis and structure of a type VI secretion membrane core complex. Nature.

[20] Zheng J, Ho B, Mekalanos JJ (2011). Genetic analysis of anti-amoebae and anti-bacterial activities of the type VI secretion system in *Vibrio cholerae*. PLoS ONE.

[21] MacIntyre DL, Miyata ST, Kitaoka M, Pukatzki S (2010). The *Vibrio cholerae* type VI secretion system displays antimicrobial properties. Proc. Natl Acad. Sci. USA.

[22] LeRoux M (2015). Kin cell lysis is a danger signal that activates antibacterial pathways of *Pseudomonas aeruginosa*. Elife.

[23] Dong TG, Ho BT, Yoder-Himes DR, Mekalanos JJ (2013). Identification of T6SS-dependent effector and immunity proteins by Tn-seq in *Vibrio cholerae*. Proc. Natl Acad. Sci. USA.

[24] Kaper JB, Morris JG, Levine MM (1995). Cholera. Clin. Microbiol. Rev..

[25] Pradhan S, Baidya AK, Ghosh A, Paul K, Chowdhury R (2010). The El Tor biotype of *Vibrio cholerae* exhibits a growth advantage in the stationary phase in mixed cultures with the classical biotype. J. Bacteriol..

[26] Bachmann V (2015). Bile salts modulate the mucin-activated type VI secretion system of pandemic *Vibrio cholerae*. PLoS Negl. Trop. Dis..

[27] Bernardy EE, Turnsek MA, Wilson SK, Tarr CL, Hammer BK (2016). Diversity of clinical and environmental isolates of *Vibrio cholerae* in natural transformation and contact-dependent bacterial killing indicative of type VI secretion system activity. Appl. Environ. Microbiol..

[28] Unterweger D (2012). Constitutive type VI secretion system expression gives *Vibrio cholerae* intra- and interspecific competitive advantages. PLoS ONE.

[29] Vettiger A, Basler M (2016). Type VI secretion system substrates are transferred and reused among sister cells. Cell.

[30] Borgeaud S, Metzger LC, Scrignari T, Blokesch M (2015). The type VI secretion system of *Vibrio cholerae* fosters horizontal gene transfer. Science.

[31] Ringel PD, Hu D, Basler M (2017). The role of type VI secretion system effectors in target cell lysis and subsequent horizontal gene transfer. Cell Rep..

[32] Thomas J, Watve SS, Ratcliff WC, Hammer BK (2017). Horizontal gene transfer of functional type VI killing genes by natural transformation. mBio.

[33] Ho BT, Basler M, Mekalanos JJ (2013). Type 6 secretion system-mediated immunity to type 4 secretion system-mediated horizontal gene transfer. Science.

[34] Basler M, Ho B, Mekalanos J (2013). Tit-for-tat: type VI secretion system counterattack during bacterial cell-cell interactions. Cell.

[35] Metcalf WW (1996). Conditionally replicative and conjugative plasmids carrying lacZ alpha for cloning, mutagenesis, and allele replacement in bacteria. Plasmid.

[36] Glocke I, Meyer M (2017). Extending the spectrum of DNA sequences retrieved from ancient bones and teeth. Genome Res..

[37] Gaspar JM (2018). NGmerge: merging paired-end reads via novel empirically-derived models of sequencing errors. BMC Bioinforma..

[38] Li H, Durbin R (2009). Fast and accurate short read alignment with Burrows–Wheeler transform. Bioinformatics.

[39] Picard Toolkit. Broad Institute, GitHub Repository. http://broadinstitute.github.io/picard/ (Broad Institute, 2019).

[40] Walker BJ (2014). Pilon: an integrated tool for comprehensive microbial variant detection and genome assembly improvement. PLoS ONE.

[41] Jónsson H, Ginolhac A, Schubert M, Johnson PL, Orlando L (2013). mapDamage2.0: fast approximate Bayesian estimates of ancient DNA damage parameters. Bioinformatics.

[42] Seemann T (2014). Prokka: rapid prokaryotic genome annotation. Bioinformatics.

[43] Page AJ (2015). Roary: rapid large-scale prokaryote pan genome analysis. Bioinformatics.

[44] Page AJ (2016). SNP-sites: rapid efficient extraction of SNPs from multi-FASTA alignments. Microb. Genomics.

[45] Posada D (2008). jModelTest: phylogenetic model averaging. Mol. Biol. Evol..

[46] Stöver BC, Müller KF (2010). TreeGraph 2: combining and visualising evidence from different phylogenetic analyses. BMC Bioinforma..

[47] Edgar RC (2004). MUSCLE: multiple sequence alignment with high accuracy and high throughput. Nucleic Acids Res..

[48] Darling AE, Mau B, Perna NT (2010). ProgressiveMauve: multiple genome alignment with gene gain, loss and rearrangement. PLoS ONE.

[49] Stamatakis A (2014). RAxML version 8: a tool for phylogenetic analysis and post-analysis of large phylogenies. Bioinformatics.

[50] Thelin KH, Taylor RK (1996). Toxin-coregulated pilus, but not mannose-sensitive hemagglutinin, is required for colonization by *Vibrio cholerae* O1 El Tor biotype and O139 strains. Infect. Immun..

[51] Zinnaka Y, Carpenter CJ (1972). An enterotoxin produced by noncholera vibrios. Johns Hopkins Med. J..

[52] Dziejman M (2005). Genomic characterisation of non-O1, non-O139 *Vibrio cholerae* reveals genes for a type III secretion system. Proc. Natl Acad. Sci. USA.

[53] Chun J (2009). Comparative genomics reveals mechanism for short-term and long-term clonal transitions in pandemic *Vibrio cholerae*. Proc. Natl Acad. Sci. USA.

