# Author Correction: Type VI secretion system mutations reduced competitive fitness of classical *Vibrio cholerae* biotype

**DOI:** 10.1038/s41467-022-28572-6

**Published:** 2022-02-11

**Authors:** Benjamin Kostiuk, Francis J. Santoriello, Laura Diaz-Satizabal, Fabiana Bisaro, Kyung-Jo Lee, Anna N. Dhody, Daniele Provenzano, Daniel Unterweger, Stefan Pukatzki

**Affiliations:** 1 Department of Medical Microbiology and Immunology, 6-020 Katz Group Centre, University of Alberta, Edmonton, AB T6G 2S2 Canada; 2 Department of Immunology & Microbiology, University of Colorado Denver Anschutz Medical Campus, 13001 E 17th Pl, Aurora, CO 80045 USA; 3 Department of Biology, The City College of New York, 160 Convent Avenue, New York, NY 10031 USA; 4 Mütter Research Institute, The College of Physicians of Philadelphia, Philadelphia, PA 19103 USA; 5 Department of Biology, University of Texas Rio Grande Valley, One West University Blvd, Brownsville, TX 78520 USA; 6 Max-Planck Institute for Evolutionary Biology, August-Thienemann-Straße 2, 24306 Plön, Germany; 7 Institute for Experimental Medicine, Kiel University, Michaelisstraße 5, 24105 Kiel, Germany

**Keywords:** Bacterial evolution, Bacterial genomics, Pathogens, Microbial ecology

Correction to: *Nature Communications* 10.1038/s41467-021-26847-y, published online 09 November 2021.

The original version of this Article contained errors in Fig. 4A, in which the country of isolation for the following *Vibrio cholerae* strains was incorrectly reported as Bangladesh: A46, A51, A111, A57, A61, A49, A103 and A279. The correct origins of the strains are, respectively: unknown, Egypt, unknown, India, India, unknown, unknown and Sweden. This has been corrected in both the PDF and HTML versions of the Article.

