# Supplementary material for: Type VI secretion system mutations reduced competitive fitness of classical Vibrio cholerae biotype
Source: Nat Commun. 2021 Nov 9;12:6457. doi: 10.1038/s41467-021-26847-y (PMC8578542; doi:10.1038/s41467-021-26847-y)
Supplement: Supplementary file 1 — Supplementary information. [file 41467_2021_26847_MOESM1_ESM.pdf]

## Supplementary Figures

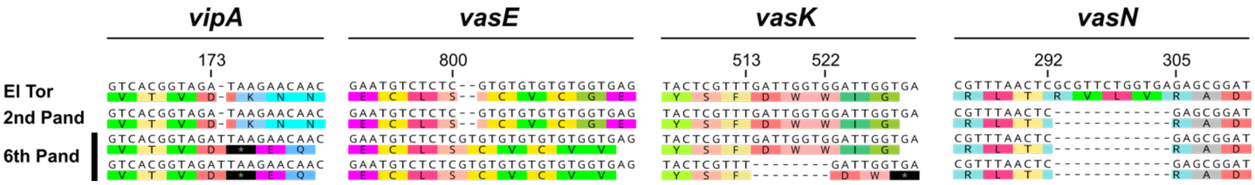


**Supplementary Figure 1. Pandemic classical *V. cholerae* strains harbour frameshift mutations in their T6SS gene clusters**. Classical *V. cholerae* encode the same frameshift mutations in the large T6SS gene cluster absent in 7th pandemic El Tor strains (Figure 4A). Shown from a MAUVE alignment are the collapsed sequences of twenty-one internal *vipA* nucleotides starting at position 163 and twenty-seven *vasE* nucleotides starting at position 790. Premature stop codons halt translation at amino acid position 66 instead of 169 for VipA (66/169), while the frame shift in *vasE* leads to a truncation at amino acid 302 (302/445) (not shown here). The same 12 nucleotide deletion in *vasN* is present in all classical but no El Tor strains examined here. In contrast, the 8 nucleotide *vasK* deletion that leads to a frameshift nonsense mutation at residue 174 is shared among classical *V. cholerae* with the exception of strains PA1849 and M29 which genotype matches that of El Tor strains.

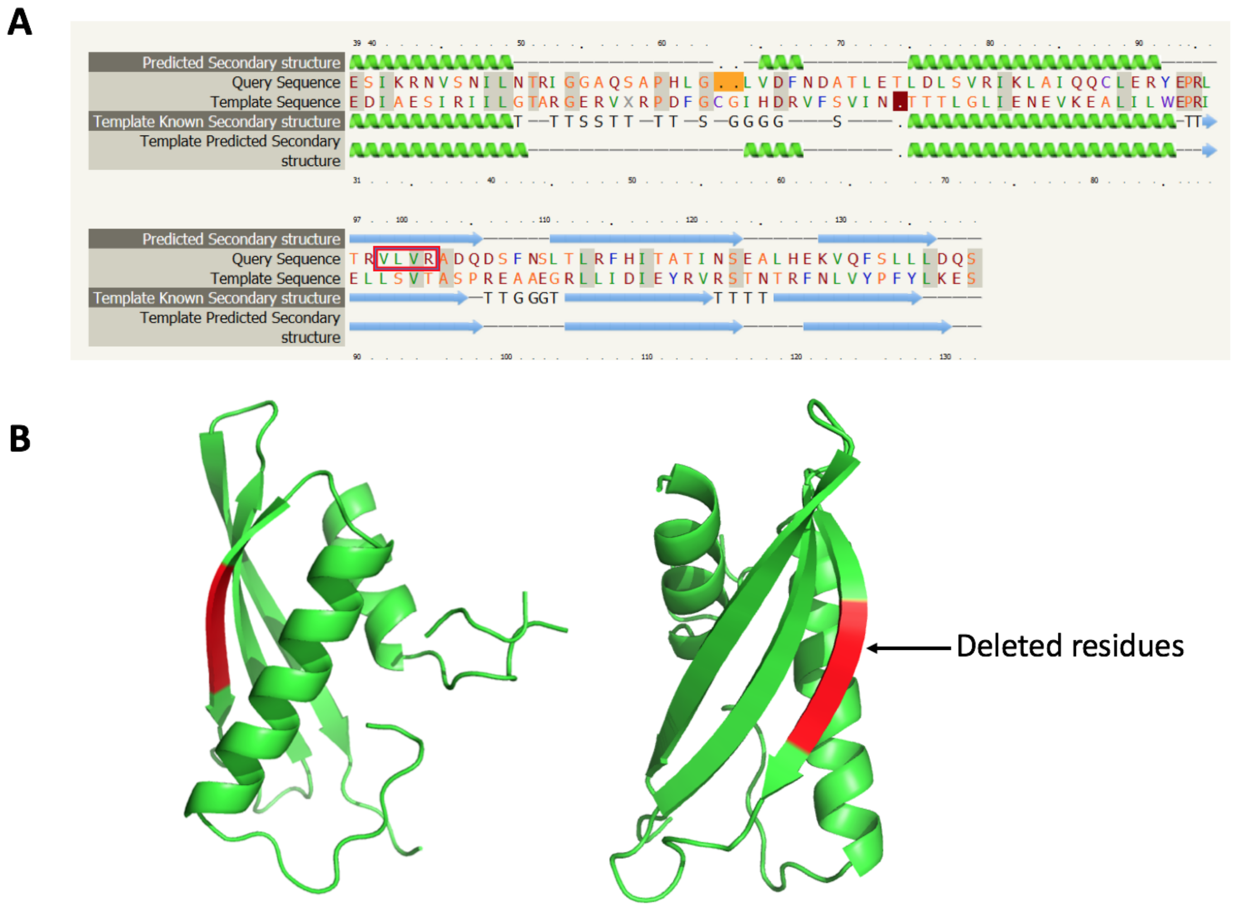


**Supplementary Figure 2. In-frame deletion in VCA0109 affects VasN secondary structure.** The amino acid sequence from the VCA0109 allele in C6706 was submitted for PHYRE2 analysis to predict secondary and tertiary structures [39]. High sequence homology with the gp27-like lysozyme allowed building this model with 97% confidence [40]. *(A)* Protein alignment of query (VCA0109) and template (gp27-like lysozyme). Beneath and above the sequence is the secondary structure. Green spirals represent alpha helices and blue arrows represent beta sheets. The red box highlights the 4 amino acids deleted in 2nd and 6th pandemic strains. *(B)* A 3-dimensional representation of VasN with the 4-amino acid deletion in 2nd and 6th pandemic strains labelled and shown in red.

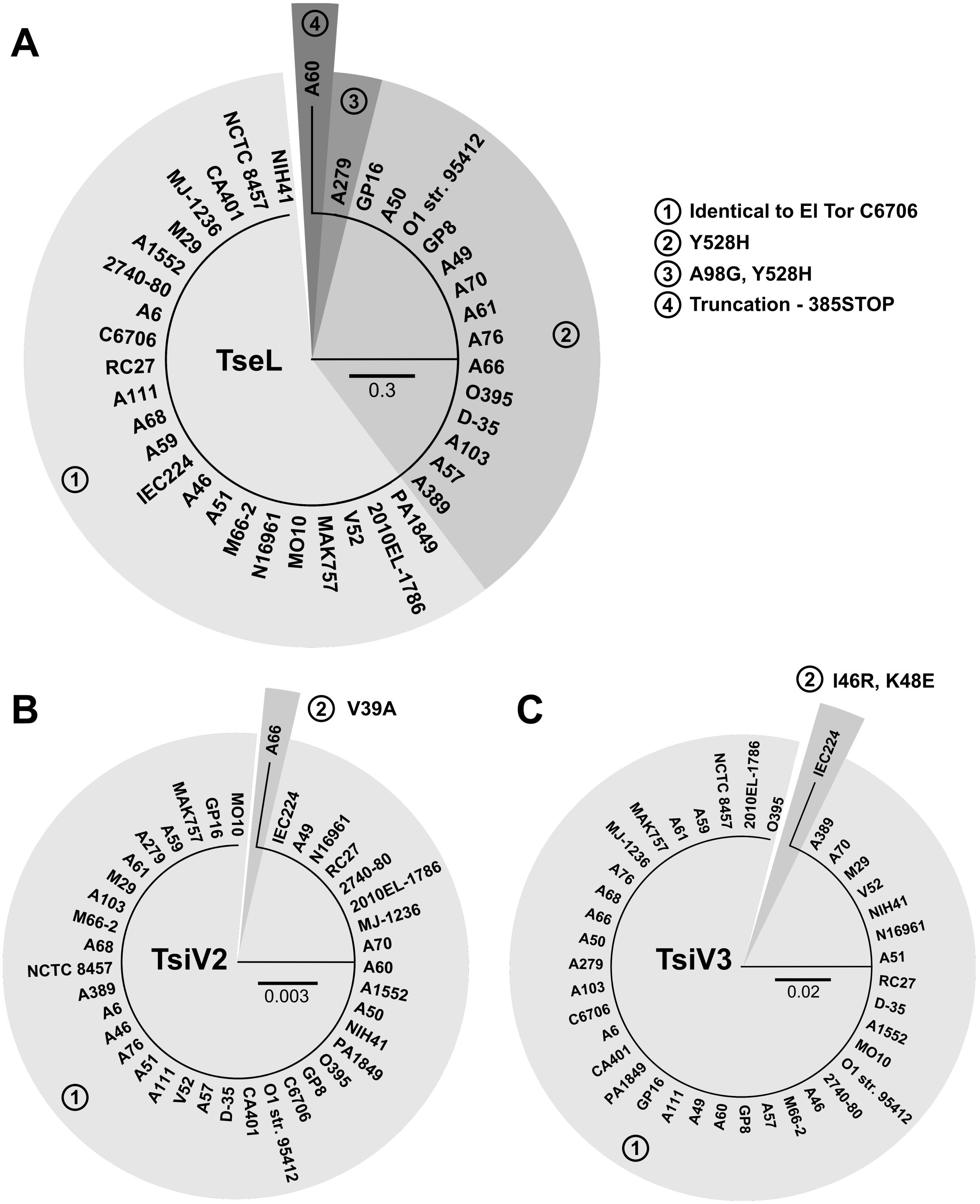


**Supplementary Figure 3. Divergence of TseL and conservation of TsiV2 and TsiV3 in classical strains.** *(A)* A radial tree of the TseL protein from the strains analysed in this study. TseL can be broken into four alleles with TseL from all El Tor strains and 10 classical strains grouping together (1), 14 classical strains carrying a Tyr528His amino

acid substitution (2), one classical strain carrying an additional Ala98Gly mutation (3), and one classical strain with a unique truncation (4). *(B)* TsiV2 and *(C)* TsiV3 from classical strains are identical to the corresponding proteins of strain C6706 with the exception of A66 (TsiV2, Val39Ala). One El Tor strain (IEC224) carries a divergent TsiV3. Radial trees with strains that harbour the analysed sequences of TsiV2 and TsiV3, respectively, are shown.

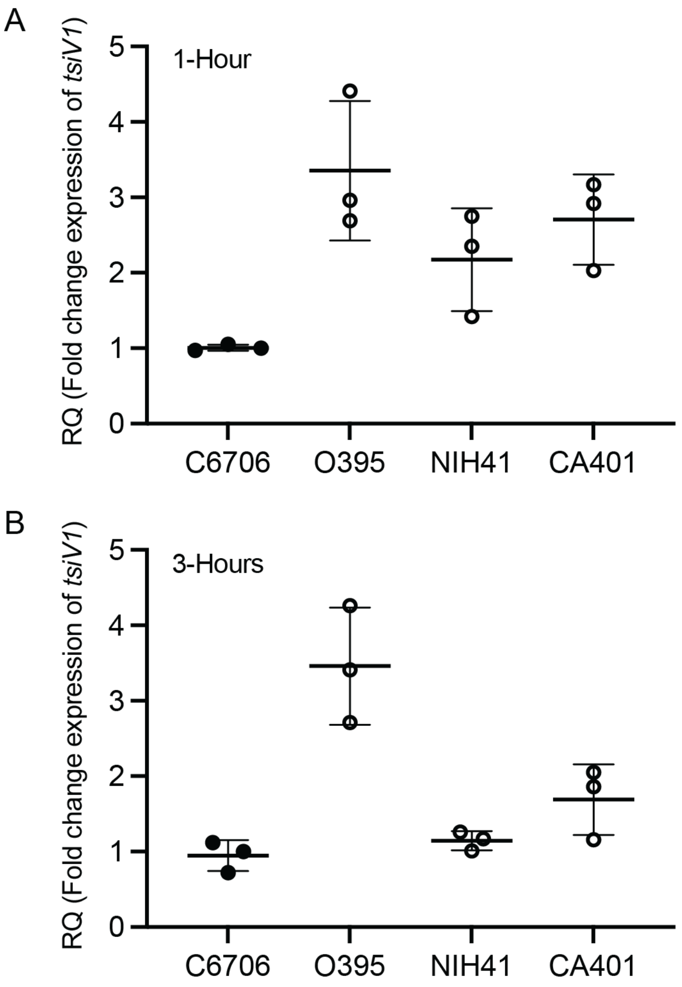


**Supplementary Figure 4. Transcription of *tsiV1* in select classical *V. cholerae* strains** (open circles) compared to the El Tor strain C6706 (filled circles). Relative quantification (RQ) of *tsiV1* transcription compared to 16S was determined for each strain, and the relative RQ values of each strain were normalised to the average relative RQ value of C6706. *(A)* Analysis of strains grown on an LB agar plate for 1 h. Unpaired t-tests were performed with Welch's correction comparing the three classical strains (O395, NIH41, and CA401) against C6706. The resulting p-values were 0.048, 0.0971 and 0.0385 respectively. *(B)* Analysis of strains grown on an LB agar plate for 3 h. Unpaired t-tests were performed with Welch's correction comparing the three classical strains (O395, NIH41, and CA401) against C6706. The resulting p-values were 0.024, 0.2378 and 0.0945 respectively. The horizontal bars represent the mean ± standard deviation of the experiments. The arithmetic mean ± standard deviation of n=2 independent experiments, each performed in triplicates, is shown. Source data are provided as a Source Data file.

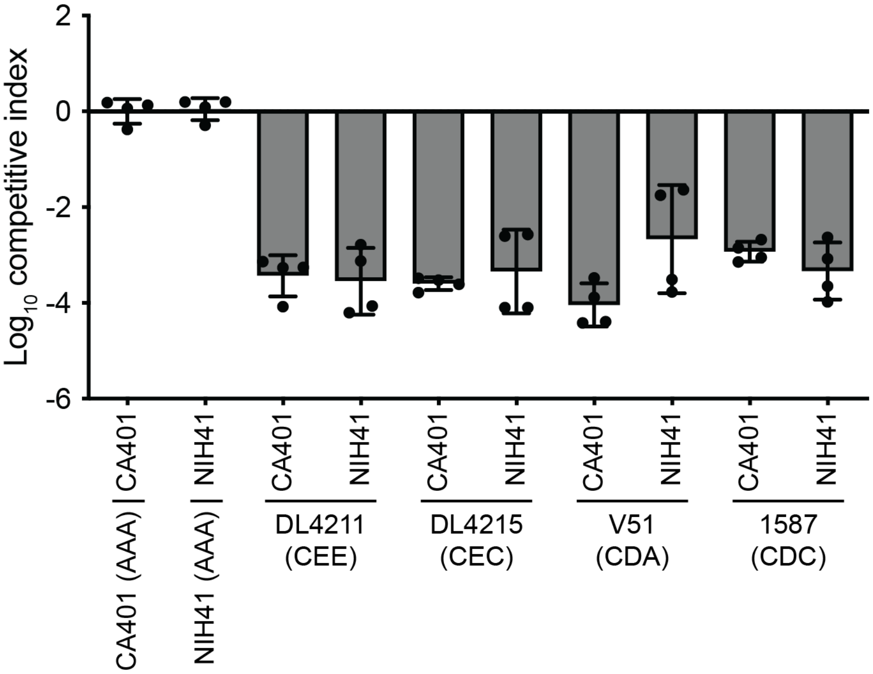


**Supplementary Figure 5. Classical 6th pandemic strains are outcompeted by non-AAA *V. cholerae*.** Classical strains CA401 and NIH41 were exposed to environmental *V. cholerae* (compatibility group memberships shown in parentheses) at a ratio of 1:1 and incubated for 4 h at 37ºC. The competitive index was calculated by dividing the ratio of CFU/mL of the 2 competing strains at t = 4 h by the ratio of the two strains at t = 0 h. The mean ± standard deviation of n=2 independent experiments, each performed in duplicates, is shown. Source data are provided as a Source Data file.

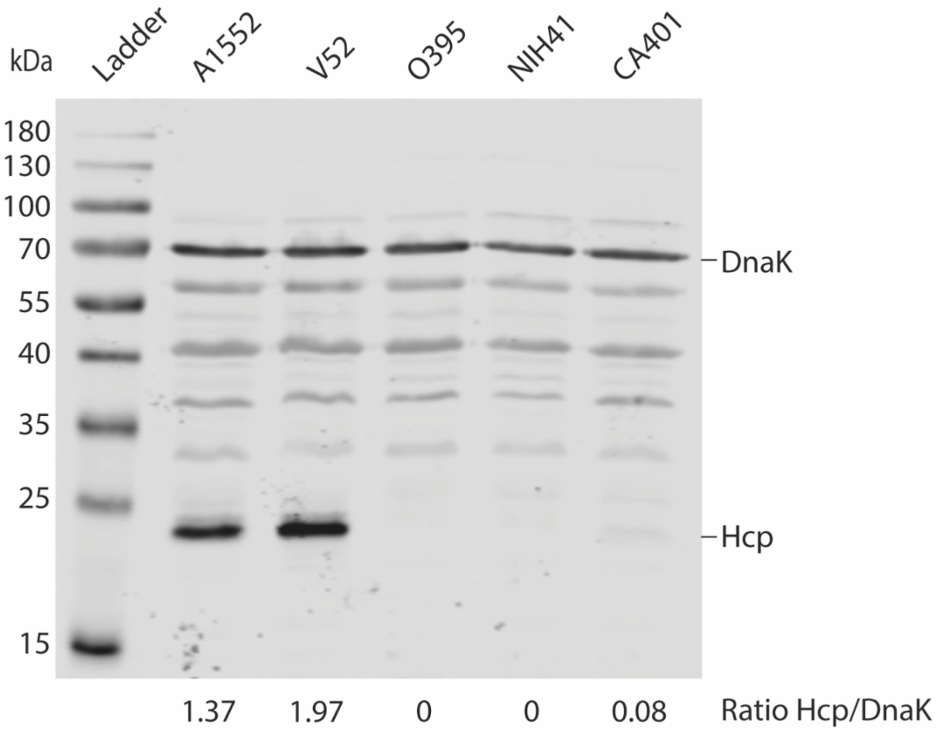


**Supplementary Figure 6. Hcp expression is reduced in classical O1 *Vibrio cholerae* strains.** El Tor A1552 and O37-serogroup strain V52 express Hcp, the principal structural component of T6SS's inner tube. No Hcp is detectable from cell pellets of classical strains O395 and NIH41 while strain CA401 produces residual Hcp levels when grown under identical conditions. This image is a chosen representative of one of four: two biological replicates split into two technical replicates each. Source data are provided as a Source Data file.

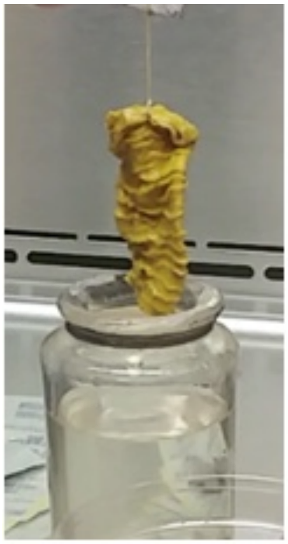

**Supplementary Figure 7. Historical Intestinal Specimen 3090.13.** Photo depicts the removal of a preserved portion of intestine from an 1849 cholera victim prior to removing a portion for this study. This specimen is part of the Mütter Museum collections.

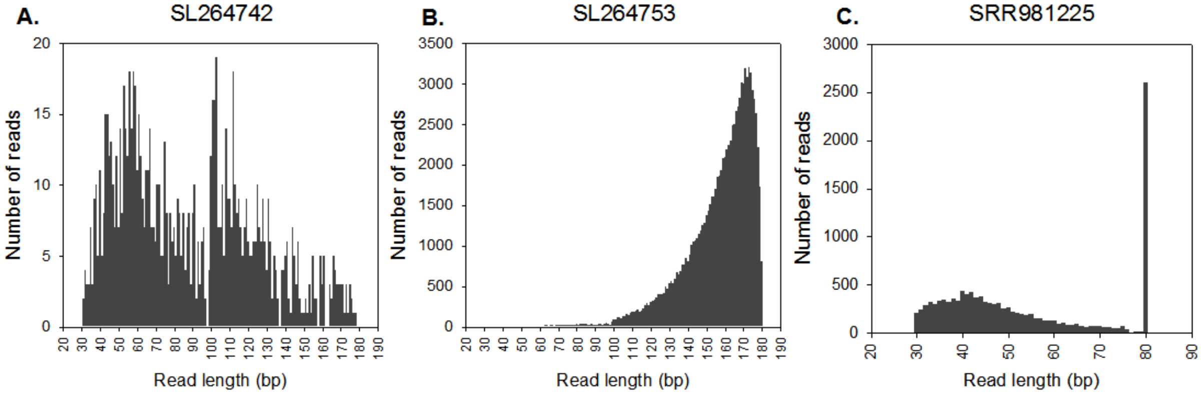


**Supplementary Figure 8. Fragment length distribution of PA1849 reads mapped to the *V. cholerae* O395 T6SS.** A. Histogram **SL264742** shows fragment length distribution of total merged reads mapped to the three T6SS clusters (not enriched). B. Histogram **SL264753** shows fragment length distribution of total merged reads enriched with T6SS booster/O395 probes and mapped to the three T6SS clusters (enriched). C. Histogram **SRR981225** shows fragment length distribution of total reads from Devault *et al.* enriched with O395 probes and mapped to the three T6SS clusters (enriched).

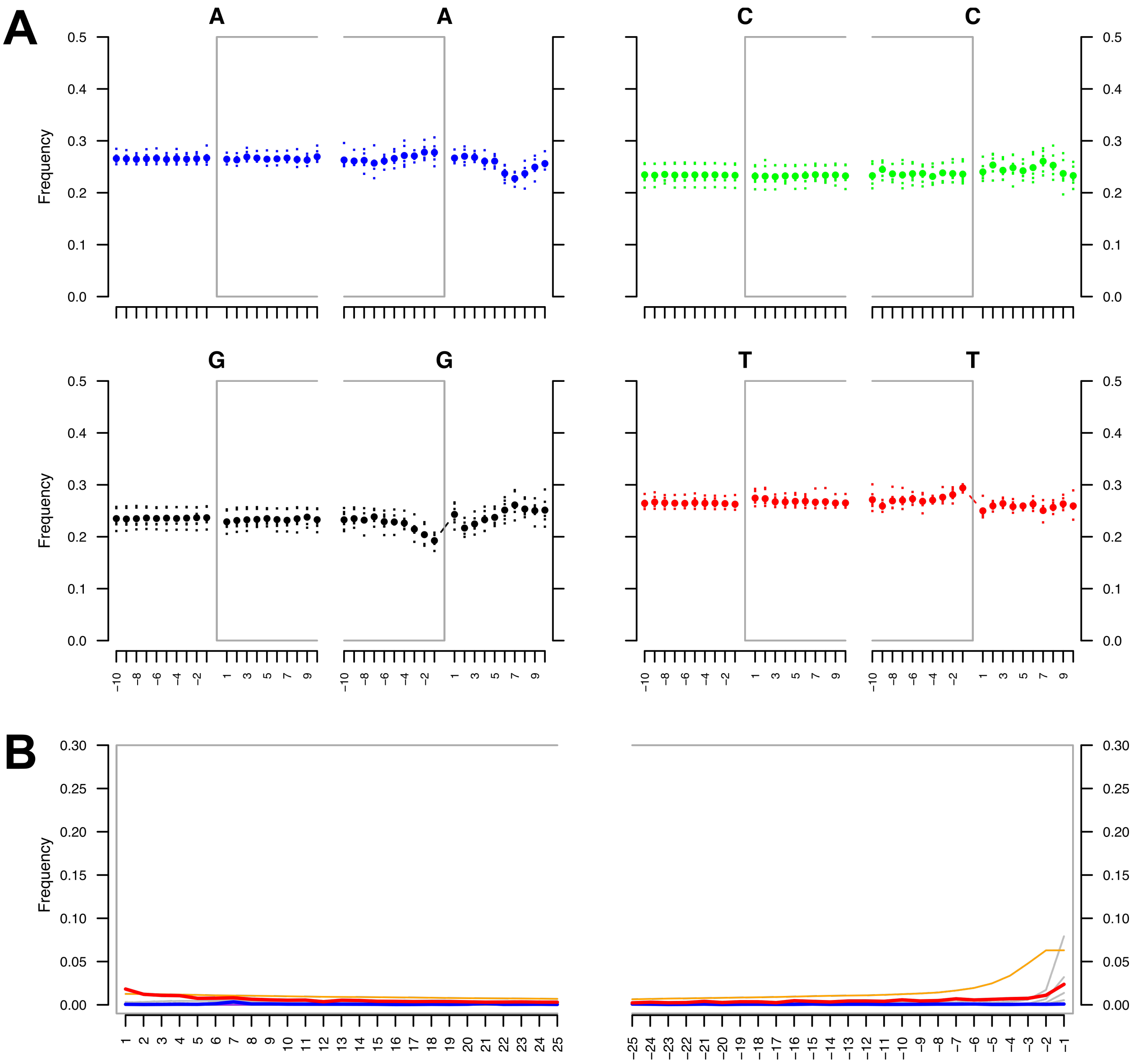


**Supplementary Figure 9. Damage profile for PA1849 reads mapped to the *V. cholerae* O395 T6SS.** (*A*) Base frequency plots representing the frequency of each nucleotide inside (within the grey brackets) and outside of each mapped read. Within the grey brackets, positive numbers represent nucleotide positions from the 5’ end of the reads, and negative numbers represent nucleotide positions from the 3’ end of the reads. Outside of the grey brackets, negative numbers represent nucleotide positions upstream of the 5’ end of the reads, and positive numbers represent nucleotide positions downstream of the 3’ end of the read. (*B*) Plot representing the frequency of position-specific substitutions for the mapped reads. The red line represents C>T substitutions. The blue line represents G>A substitutions. The orange line represents the frequency of soft-clipping. Grey lines represent all other substitutions. Positive numbers represent nucleotide positions from the 5’ end of the reads, and negative numbers represent nucleotide positions from the 3’ end of the reads. A low frequency of C>T substitutions is seen at each end of the mapped reads. This damage pattern is

non-canonical for ancient DNA likely due to the young age and preservation of the sample (Supplementary Figure 7), as noted by Devault *et al.*, 2014[1].

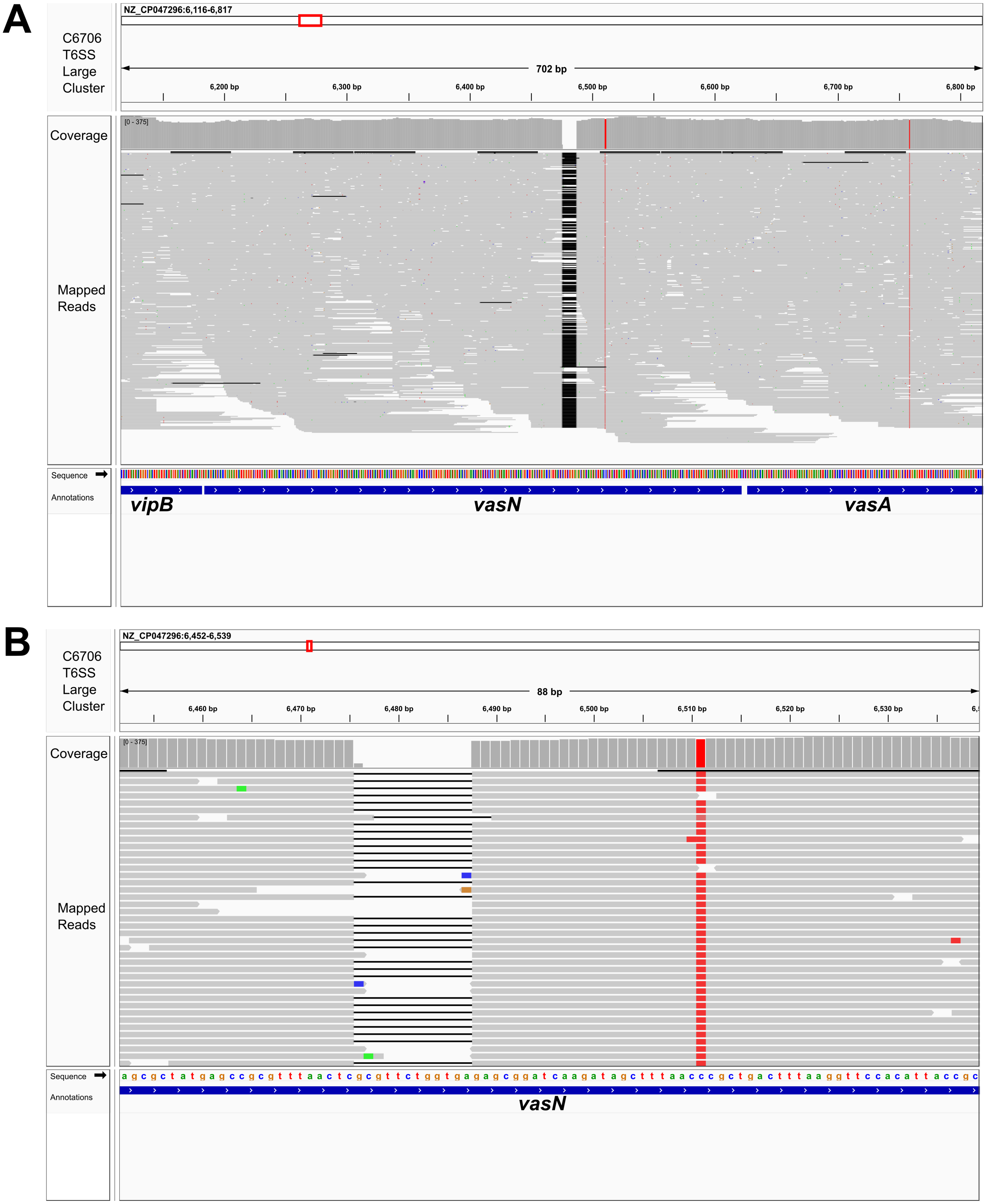


**Supplementary Figure 10. PA1849 reads carry the 8 bp *vasN* deletion.** (*A*) Expanded and (*B*) zoomed-in coverage plots and read pile-up at the region of the *V. cholerae* C6706 T6SS Large cluster encoding VasN. Colored bases in the read pile-up track represent mismatches to the reference sequence. Colored bases in the coverage

track represent consensus variants. Black lines represent gaps spanned by individual aligned reads. Plots represent a lack of read coverage at the proposed 8-bp *vasN* deletion with many reads bridging the deleted region.

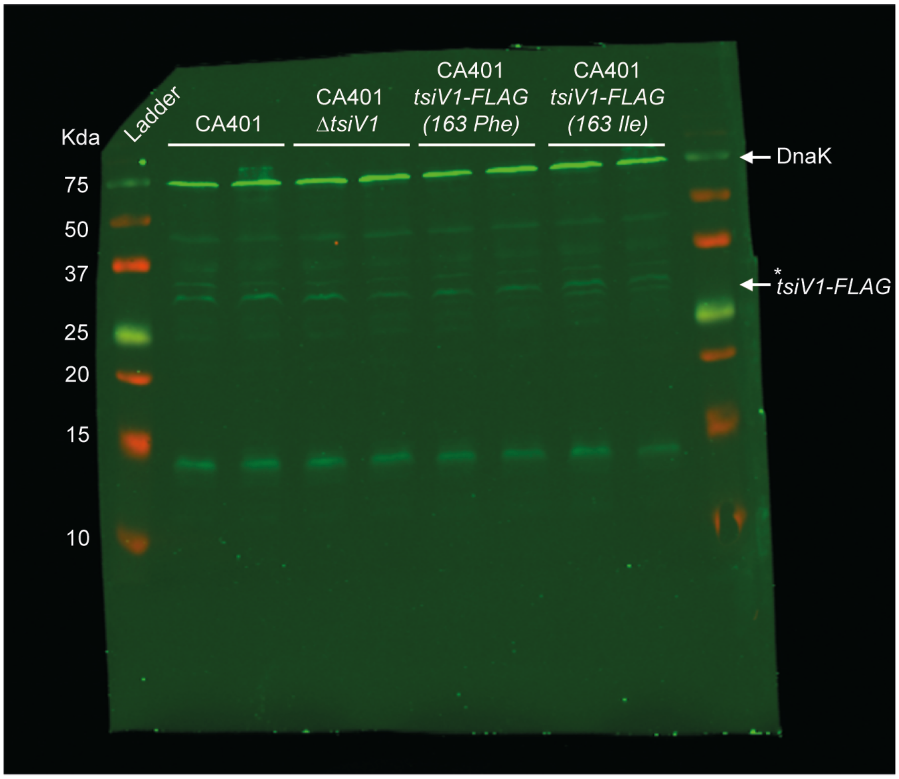


**Supplementary Figure 11. Uncropped and unprocessed scan of blot from Figure 3D.** Quantification is shown in source data file.
