# Supplementary material for: Type VI secretion system mutations reduced competitive fitness of classical Vibrio cholerae biotype
Source: Nat Commun. 2021 Nov 9;12:6457. doi: 10.1038/s41467-021-26847-y (PMC8578542; doi:10.1038/s41467-021-26847-y)
Supplement: Supplementary file 2 — Reporting summary. [file 41467_2021_26847_MOESM2_ESM.pdf]

nature portfolio

Corresponding author(s): Stefan Pukatzki

Last updated by author(s): Sep 6, 2021

Reporting Summary

Nature Portfolio wishes to improve the reproducibility of the work that we publish. This form provides structure for consistency and transparency in reporting. For further information on Nature Portfolio policies, see our Editorial Policies and the Editorial Policy Checklist.

Statistics

For all statistical analyses, confirm that the following items are present in the figure legend, table legend, main text, or Methods section.

n/a	Confirmed	
☐	☒	The exact sample size (*n*) for each experimental group/condition, given as a discrete number and unit of measurement
☐	☒	A statement on whether measurements were taken from distinct samples or whether the same sample was measured repeatedly
☐	☒	The statistical test(s) used AND whether they are one- or two-sided<br>*Only common tests should be described solely by name; describe more complex techniques in the Methods section.*
☒	☐	A description of all covariates tested
☐	☒	A description of any assumptions or corrections, such as tests of normality and adjustment for multiple comparisons
☐	☒	A full description of the statistical parameters including central tendency (e.g. means) or other basic estimates (e.g. regression coefficient) AND variation (e.g. standard deviation) or associated estimates of uncertainty (e.g. confidence intervals)
☐	☒	For null hypothesis testing, the test statistic (e.g. *F*, *t*, *r*) with confidence intervals, effect sizes, degrees of freedom and *P* value noted<br>*Give P values as exact values whenever suitable.*
☒	☐	For Bayesian analysis, information on the choice of priors and Markov chain Monte Carlo settings
☒	☐	For hierarchical and complex designs, identification of the appropriate level for tests and full reporting of outcomes
☒	☐	Estimates of effect sizes (e.g. Cohen's *d*, Pearson's *r*), indicating how they were calculated

Our web collection on statistics for biologists contains articles on many of the points above.

Software and code

Policy information about availability of computer code

Data collection	Quantitative PCR data were collected in Bio-Rad CFX Manager 3.1.
Data analysis	Genomic FASTA files for tree building were obtained from the PATRIC database or NCBI and annotated using Prokka (v1.12). A core genome was extracted from Prokka-output GFF3 files using Roary (v3.11.2). The core genome alignment was reduced to only loci harboring polymorphisms using SNP-sites (v2.4.1). A Maximum Likelihood phylogenetic tree was built using the RAxML (v8.2.12) GTR model. Model was selected using jModelTest (v0.1.6). Phylogenetic trees were visualized from RAxML-generated newick files using TreeGraph 2 (v2.15.0-887 beta). Alignments of amino acid sequences were performed with MUSCLE (v3.8.425) and alignments of the nucleotide sequences were performed using the progressive MAUVE algorithm (v1.1.1) in Geneious (Geneious Prime v2019.0.4). Aligned sequences were analyzed using RAxML (nucleotide model: GTR Gamma, Algorithm: Rapid hill-climbing). SRA read files were aligned to T6SS clusters to generate consensus sequences and identify polymorphisms in Geneious (Geneious Prime v2019.0.4).

For manuscripts utilizing custom algorithms or software that are central to the research but not yet described in published literature, software must be made available to editors and reviewers. We strongly encourage code deposition in a community repository (e.g. GitHub). See the Nature Portfolio guidelines for submitting code & software for further information.

Data

Policy information about availability of data

All manuscripts must include a data availability statement. This statement should provide the following information, where applicable:

- Accession codes, unique identifiers, or web links for publicly available datasets
- A description of any restrictions on data availability
- For clinical datasets or third party data, please ensure that the statement adheres to our policy

The authors declare that all the data supporting the findings of this study are available within the paper and its supplementary information files. All genomes analysed in this study are publicly available from the PATRIC (https://www.patricbrc.org/) and NCBI RefSeq (https://www.ncbi.nlm.nih.gov/refseq/) databases. All sequence reads analysed in this study are publicly available from the NCBI SRA database (https://www.ncbi.nlm.nih.gov/sra). Reads generated in this study are available from the NCBI SRA database (PRJNA767369).

Field-specific reporting

Please select the one below that is the best fit for your research. If you are not sure, read the appropriate sections before making your selection.

☒ Life sciences ☐ Behavioural & social sciences ☐ Ecological, evolutionary & environmental sciences

For a reference copy of the document with all sections, see nature.com/documents/nr-reporting-summary-flat.pdf

Life sciences study design

All studies must disclose on these points even when the disclosure is negative.

Sample size	No calculations were performed to determine sample size. Experiments were performed in biological triplicate based on previous experience with similar experiments and on previously published research using similar methods (Rajanna et al. J. Bacteriol. 2003, 10.1128/jb.185.23.6893-6901.2003; Murphy & Boyd et al. J. Bacteriol. 2008, 10.1128/JB.00562-07; Almagro-Moreno et al. BMC Microbiology, 10.1186/1471-2180-10-306; Carpenter et al. J. Bacteriol. 2016, 10.1128/JB.00704-15; Labbate et al. Sci. Rep. 2016, 10.1128/JB.00704-15;).
Data exclusions	No data were excluded from the manuscript.
Replication	All experiments were performed with independent replicates as described in the figure legends.
Randomization	Randomization is not applicable to this study as it uses bacterial strains. Sample groups consist of replicates taken from a stock culture on multiple, independent occasions. This study does not involve human participants or animal models, and thus does not require randomization.
Blinding	When experiments required manual counts of colony forming units from different treatments (Fig. 2, Fig. 3c,d, Fig. S5), CFUs were counted blind to strain and selective antibiotics to avoid bias in the counts. Identification of nucleotide/amino acid polymorphisms was performed blinded to the strain identity and biotype of each sample (Fig. 4a).

Reporting for specific materials, systems and methods

We require information from authors about some types of materials, experimental systems and methods used in many studies. Here, indicate whether each material, system or method listed is relevant to your study. If you are not sure if a list item applies to your research, read the appropriate section before selecting a response.

Materials & experimental systems

n/a	Involved in the study	
☐	☒	Antibodies
☒	☐	Eukaryotic cell lines
☒	☐	Palaeontology and archaeology
☒	☐	Animals and other organisms
☐	☒	Human research participants
☒	☐	Clinical data
☒	☐	Dual use research of concern

Methods

n/a	Involved in the study	
☒	☐	ChIP-seq
☒	☐	Flow cytometry
☒	☐	MRI-based neuroimaging

Antibodies

Antibodies used	DnaK: Enzo ADI-SPA-880F; FLAG: Sigma Aldrich F1804; Secondary AB for DnaK and FLAG: LICOR 926-32210; secondary AB for Hcp: :ICOR 925-68071; for Hcp antibody, please see PNAS 2007, 104(39): 15508-15513,

Validation	*Describe the validation of each primary antibody for the species and application, noting any validation statements on the manufacturer's website, relevant citations, antibody profiles in online databases, or data provided in the manuscript.*

Human research participants

Policy information about studies involving human research participants

Population characteristics	This study involves tissue collected from a single intestinal sample (specimen 3090.13) taken from a cholera patient in 1849 and archived at the Mutter Museum in Philadelphia, PA.
Recruitment	The Mutter Museum was contacted, and they agreed to donate a portion of the sample for this study.
Ethics oversight	This study was approved by the Mutter Museum and the University of Colorado.

Note that full information on the approval of the study protocol must also be provided in the manuscript.
